# Interaction between [(η^6^-*p*-cym)M(H_2_O)_3_]^2+^ (M^II^ = Ru, Os) or [(η^5^-Cp*)M(H_2_O)_3_]^2+^ (M^III^ = Rh, Ir) and Phosphonate Derivatives of Iminodiacetic Acid: A Solution Equilibrium and DFT Study

**DOI:** 10.3390/molecules28031477

**Published:** 2023-02-03

**Authors:** Linda Bíró, Botond Tóth, Norbert Lihi, Etelka Farkas, Péter Buglyó

**Affiliations:** Department of Inorganic & Analytical Chemistry, Faculty of Science and Technology, University of Debrecen, Egyetem tér 1, H-4032 Debrecen, Hungary

**Keywords:** IDA-based phosphonates, organoruthenium, organorhodium, solution equilibrium, complex

## Abstract

The pH-dependent binding strengths and modes of the organometallic [(η^6^-*p*-cym)M(H_2_O)_3_]^2+^ (M^II^ = Ru, Os; *p*-cym = 1-methyl-4-isopropylbenzene) or [(η^5^-Cp*)M(H_2_O)_3_]^2+^ (M^III^ = Rh, Ir; Cp* = pentamethylcyclopentadienyl anion) cations towards iminodiacetic acid (H_2_Ida) and its biorelevant mono- and diphosphonate derivatives N-(phosphonomethyl)-glycine (H_3_IdaP) and iminodi(methylphosphonic acid) (H_4_Ida2P) was studied in an aqueous solution. The results showed that all three of the ligands form 1:1 complexes via the tridentate (O,N,O) donor set, for which the binding mode was further corroborated by the DFT method. Although with IdaP^3−^ and Ida2P^4−^ in mono- and bis-protonated species, where H^+^ might also be located at the non-coordinating N atom, the theoretical calculations revealed the protonation of the phosphonate group(s) and the tridentate coordination of the phosphonate ligands. The replacement of one carboxylate in Ida^2−^ by a phosphonate group (IdaP^3−^) resulted in a significant increase in the stability of the metal complexes; however, this increase vanished with Ida2P^4−^, which was most likely due to some steric hindrance upon the coordination of the second large phosphonate group to form (5 + 5) joined chelates. In the phosphonate-containing systems, the neutral 1:1 complexes are the major species at pH 7.4 in the millimolar concentration range that is supported by both NMR and ESI-TOF-MS.

## 1. Introduction

Phosphonic acids containing C–P(O)(OH)_2_ or C–P(O)(OR)_2_ (R = alkyl, aryl) groups, or their deprotonated forms, phosphonates, are organic compounds that are capable of coordinating various metal ions. They have a wide range of applications, such as in (pro)drugs in medicine, among others. The presence of strong P–C bonds also makes them resistant to hydrolysis, allowing their use in the treatment of disorders of the calcium metabolism and bone diseases [1,2,3,4,5,6].

Aminophosphonic acids represent an important subclass of phosphonic acids and are considered as structural analogues of the corresponding amino acids. The ability of an amino group to obtain a –NH–CH_2_–P(O)(OR)_2_ scaffold increases the denticity and, therefore, the metal binding capability of phosphonates [7]. Despite the structural similarity, aminophosphonic acids and amino acids differ considerably in their geometry, their size, their charge, and their acidity. The tetrahedral –P(O)(OH)_2_ function has a larger steric demand and an increased basicity compared to the planar –COOH group. In aminophosphonates, the deprotonation of the -OH units in the diprotic phosphonic group takes place in the pH-ranges of 0.5–1.5 and 5.0–6.0, respectively, while the proton loss of –COOH in amino acids occurs at pH ~ 2.0–3.0 [7,8]. Comparative solution studies with different di- and trivalent cations, Ca(II), Mg(II), Al(III), Mn(II), V(IV)O, Cu(II), and Zn(II) have revealed the formation of complexes with an increased stability by the replacement of carboxylates by phosphonates (due to the higher overall basicity of the latter); however, this effect was found to be compensated, or overcompensated, with an increasing number of phosphonates, due to their higher electrostatic and steric hindrance [9,10,11,12].

N-(phosphonomethyl)glycine (glyphosate), which is a representative of the aminophosphonic acids, is one of the most commonly used broad spectrum herbicides. Moreover, the antibiotic, antiviral, and antitumor activity of glyphosate derivatives has also been reported [7]. It has been found that the incorporation of the phosphonate/aminophosphonate functionality into clinically used drugs results in substances with increased biological activity [13,14].

Owing to their high affinity to calcified tissues, bisphosphonates can act as carrier molecules for metal ions with anticancer activity. Furthermore, the combination of these types of ligands and metal ions with anticancer potential into one molecule may provide complexes that are suitable for the therapy of both primary and metastatic tumors, which was found previously for a cisplatin-linked phosphonate complex [13].

A large number of complexes incorporating organometallic platinum group metal ions (e.g., [(η^6^-arene)M]^2+^ (M^II^ = Ru, Os) or [(η^5^-Cp*)M]^2+^ (M^III^ = Rh, Ir; Cp* = pentamethylcyclopentadienyl anion)) that exhibit promising anticancer potential with fewer side-effects compared to cisplatin have been synthesized and characterized in the solid state during the past decades [15,16,17,18,19,20,21,22,23]. In order to explore the fate of these compounds, and to obtain reliable speciation in an aqueous medium, solution equilibrium studies may provide us with help exploring the stoichiometry, the stability, and likely the binding mode of the species that are present in a solution. In this context, in the last decade, the complexation processes of several systems containing either [(η^6^-*p*-cym)Ru(H_2_O)_3_]^2+^ (*p*-cym = 1-methyl-4-isopropylbenzene) or [(η^5^-Cp*)Rh(H_2_O)_3_]^2+^ cations were explored [24,25,26,27,28,29,30]. With this type of metal ion, however, only a single publication can be found relating to any direct interaction with phosphonates. It was very recently demonstrated that glyphosate and glyphosine (N-bis(phosphonomethyl)glycine) coordinates to [(η^5^-Cp*)Ir(H_2_O)_3_]^2+^ in a tridentate manner to form stable (O,N,O) chelates. The retained piano-stool geometry of the complexes was supported by XRD. The hybrid materials that were derived from supporting the above complexes onto rutile TiO_2_ were found to be effective molecular catalysts in water oxidation [31]. In another study, aminophosphonate complexes of [(η^6^-*p*-cym)Ru]^2+^ were used as catalysts to reduce ketones, however, in this case, the phosphonate units were not in the coordination sphere of metal ion [32].

The above examples clearly represent the biological importance of the platinum group metal ion–phosphonate interactions. Therefore, the goal of the present work was to explore the role of the phosphonate functions in [(η^6^-*p*-cym)M(H_2_O)_3_]^2+^ (M^II^ = Ru, Os) or [(η^5^-Cp*)M(H_2_O)_3_]^2+^ M^III^ = Rh, Ir) binding and to determine the stoichiometry, the stability, and the most plausible structure of the complexes that are formed in an aqueous solution.

**Scheme 1:**
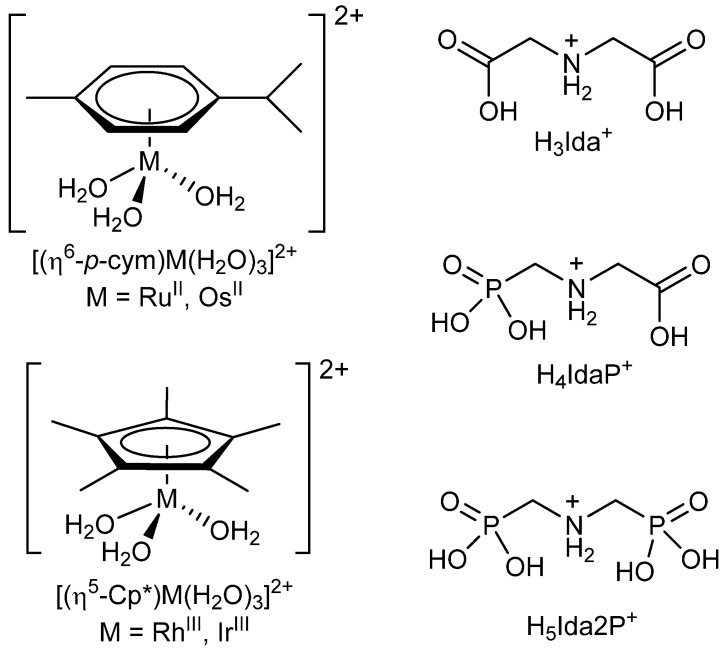
Structure of the cations and the fully protonated forms of the ligands studied.

Herein, we report on the results of a comparative solution equilibrium study on the interaction of these metal ions with iminodiacetic acid (H_2_Ida) and its phosphonic derivatives, N-(phosphonomethyl)-glycine (H_3_IdaP) and iminodi(methylphosphonic acid) (H_4_Ida2P) (Scheme 1), which were obtained by the combined use of pH-potentiometry, ^1^H NMR, and ESI-TOF MS, as well as with DFT calculations.

## 2. Results and Discussion

### 2.1. Proton Dissociation Processes of the Ligands

In order to check the purity of the ligands and to determine the exact concentration of their stock solutions, the stepwise protonation constants were determined under our experimental conditions. The evaluation of the titration curves resulted in the log*K_i_* values that are summarized in Table 1.

**Figure 1:**
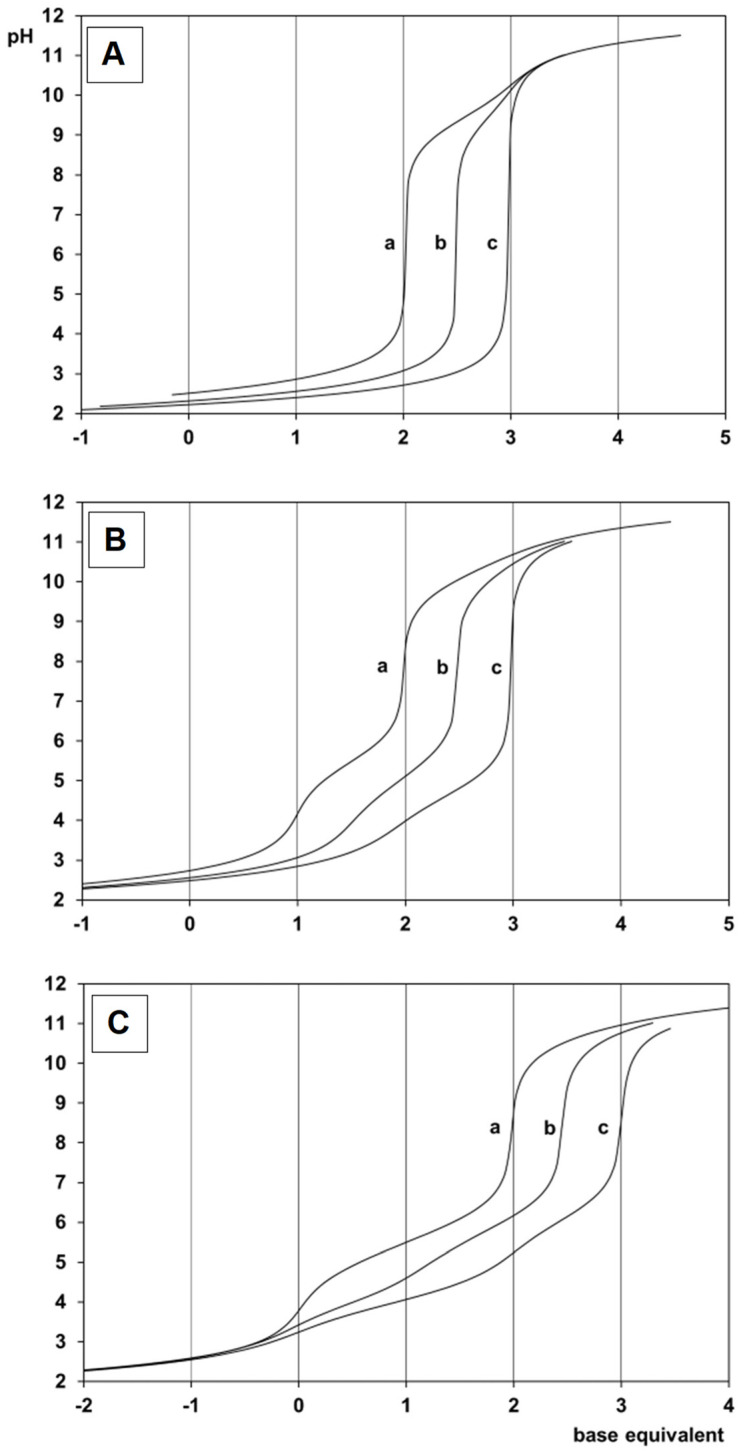
pH-potentiometric titration curves with Ida^2−^ (**A**), IdaP^3−^ (**B**), and Ida2P^4−^ (**C**) for the H^+^–ligand system (a) and [(η^5^-Cp*)Rh]^2+^–ligand systems at 1:2 (b) and 1:1 (c) metal ion to ligand ratios. Negative base equivalent refers to an excess of acid in the sample. *T* = 25.0 °C, *I* = 0.20 M KCl.

In principle, Ida^2−^ has three, IdaP^3−^ has four, while Ida2P^4−^ has five donor atoms to be protonated. Starting with the titrations from the acidic range, the appropriate curves (see Figure 1) reveal that, for H_3_Ida^+^, the deprotonation of the two carboxylic functions is complete at pH 4, while the secondary ammonium group loses its proton at around pH 9. For the phosphonic acids in the measurable pH range, only one –OH of the phosphonic group is protonated, while the other is already in its deprotonated form, due to the high acidity of that –OH group [9]. This is reflected in the titration curves. In addition to the deprotonation of the secondary ammonium group at around pH 10, at a lower pH, there is one for H_3_IdaP, while there are two equivalents base consumption for H_4_Ida2P, which indicates the deprotonation of the phosphonic functions of the ligands. The obtained log*K_i_* in Table 1 agrees well with the previously determined values, which were calculated under identical or very similar conditions [9]. The higher basicity of the imino group in IdaP^3−^, and especially in Ida2P^4−^, can be interpreted by the increased electron density on it due to the presence of an increasing number of doubly negatively charged phosphonate groups instead of the carboxylate(s).

### 2.2. Complexation Processes with the Organometallic Cations

The titration curves at 1:1 and 1:2 metal ion to ligand ratios were registered (Figure 1) and evaluated, resulting in the models and the stability constants that are summarized in Table 2. Although chloride ions have a higher affinity to coordinate to these metal ions compared to water, they cannot compete efficiently with the tridentate ligands that have been studied here. This means that in the coordination sphere of the free metal ions, chloride ions are likely present, but upon the formation of the complexes with the ligands, the chloride ions will be displaced. The slight concurring effect of the chloride ions on the complexation processes with the ligands is therefore taken into consideration by the log*β* values in Table 2, as they are conditional stability constants and are valid only under the experimental conditions that have been used. Hereafter in this paper, the [(η^6^-*p*-cym)Ru(H_2_O)_3_]^2+^, [(η^6^-*p*-cym)Os(H_2_O)_3_]^2+^, [(η^5^-Cp*)Rh(H_2_O)_3_]^2+^, and [(η^5^-Cp*)Ir(H_2_O)_3_]^2+^ ions will be referred to as [(η^6^-*p*-cym)Ru]^2+^, [(η^6^-*p*-cym)Os]^2+^, [(η^5^-Cp*)Rh]^2+^, and [(η^5^-Cp*)Ir]^2+^, respectively. 

**Figure 2:**
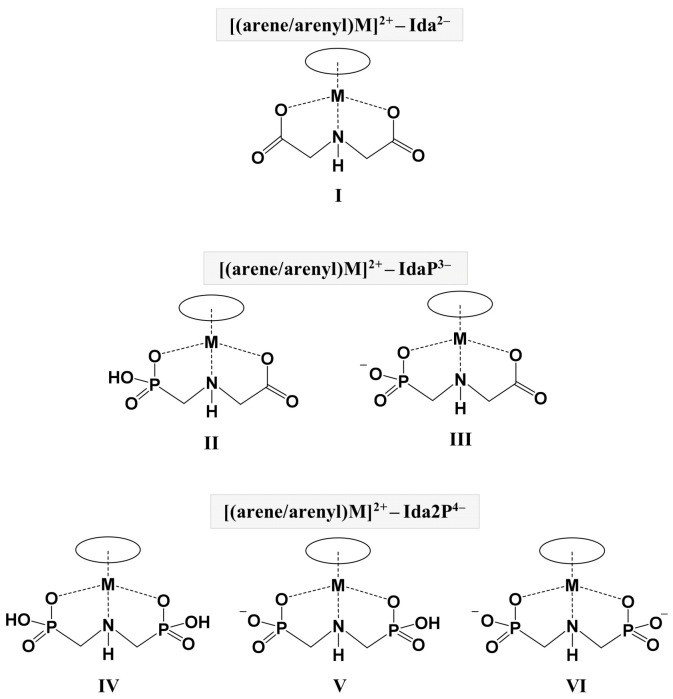
Suggested solution structures of the complexes formed in the [(arene/arenyl)M]^2+^ – Ida^2−^, IdaP^3−^ and Ida2P^4−^ systems ([(arene)M]^2+^ = [(η^6^-*p*-cym)Ru]^2+^, [(η^6^-*p*-cym)Os]^2+^; [(arenyl)M]^2+^ = [(η^5^-Cp*)Rh]^2+^, [(η^5^-Cp*)Ir]^2+^).

In the [(η^5^-Cp*)Rh]^2+^–Ida^2−^ and –IdaP^3−^ systems (Figure 1), the complex formation starts as low as pH ~ 2, but even with Ida2P^4−^ this process is significant above pH ~ 3. In all of these systems, no hydrolysis of the metal ion is indicated in the basic pH range, while the shape of the 1:2 curves reveal the lack of formation of complexes with 1:2 stoichiometry. For the phosphonates, the structured titration curves suggest the formation of various protonated complexes too. While the shape of the titration curves that were obtained with [(η^6^-*p*-cym)Ru]^2+^ are essentially the same as those with the rhodium cation (see Figure S1) for the heavier congeners, [(η^6^-*p*-cym)Os]^2+^ and [(η^5^-Cp*)Ir]^2+^, very slow complexation processes were detected; furthermore, the presence of not round base equivalents on the titration curves (see Figure S2A) refer to the partial hydrolysis of these latter metal ions during the titration. The data that are presented in Table 2 support that, in addition to [ML] (the charges of the different complexed species are omitted for clarity in the whole document, except in Table 2) with Ida^2−^, complexes with [MHL] and [MH_2_L] stoichiometry are also formed with the phosphonate derivatives in all of the systems. For the [ML] species with Ida^2−^, the tridentate coordination of the ligand is assumed as follows: besides the imino-N, the carboxylate-O donors saturate the coordination sphere of the metal ions, resulting in the formation of two joined five-membered chelates with high stability (see I in Figure 2). A comparison of the stability constants of the appropriate Ida^2−^ complexes for the ruthenium and rhodium cation reveals that the latter is ~ 1.3 logarithmic units higher than the former. This result is surprising since, in the vast majority of the cases, [(η^6^-*p*-cym)Ru]^2+^ forms more stable complexes over [(η^5^-Cp*)Rh]^2+^ with the same ligand [33,34,35]. In order to shed light on this contradiction, the reversibility of the titrations for the ruthenium-Ida^2−^ system was checked and, as a control, the rhodium–Ida^2−^ samples were also involved. For both of these systems, the titrated samples were acidified and re-titrated. The comparison of the registered curves and the evaluation of the obtained data indicated no significant differences (i.e., full reversibility) for rhodium; however, the results from the second titration of the ruthenium-Ida^2−^ sample showed remarkable differences. This can be explained by the slow complexation (formation and dissociation) processes for the ruthenium-containing system, resulting in incomplete reversibility (vide infra). Notably, for all of the ligands, the complexation processes with the organoosmium and –iridium cation were also found to be very slow. Another point to be mentioned is that any peculiarity regarding the stability of the appropriate ruthenium and –rhodium complexes with the phosphonate ligands was not observed.

For the ruthenium or –rhodium–IdaP^3−^ systems, the complexation starts with the formation of the [MHL] species. The calculated p*K*_MHL_ values (4.44 for Ru and 4.72 for Rh) may support the deprotonation of the hydrogen phosphonate group of the coordinating IdaP^3−^, thus the most likely binding mode in these species is also tridentate, although one of the phosphonate oxygens of the ligand is still protonated (see structure II in Figure 2).

Since, in principle, in [MHL] species with IdaP^3−^, the proton can either be located at the imino-NH, preventing this group from coordination, or at one of the phosphonate oxygen atoms, DFT calculations were also carried out with [(η^6^-*p*-cym)Ru]^2+^. In these studies, the geometry of the complex was optimized by considering the relativistic effect of the heavy Ru atom via the effective core potential approach, while the effect of the solvent was simulated by the use of the SMD model. Such calculations provide reliable results in the prediction of solvation free energy and in the comparison of coordination isomers. The optimized structures of the two coordination isomers are shown in Figure S3 and their Cartesian coordinates are summarized in Tables S1 and S2. For isomer **A** (see Figure S3), the ligand binds the [(η^6^-*p*-cym)Ru]^2+^ via the (O,O) donors of the carboxylate and phosphonate oxygen atoms, and the protonated imino group is not involved in the coordination. On the contrary, IdaP^3−^ coordinates through the (O,N,O) donors, and one of the phosphonate oxygen atoms is protonated in isomer **B**, in which the structure yields a more relaxed arrangement. This was further corroborated by calculating the relative energy of the isomers in terms of the Δ*G*^tot^_aq_ values. The calculation revealed that the (O,O) binding mode is less stable and the (O,N,O) coordination in [(η^6^-*p*-cym)RuH(IdaP)] is more favorable with 65.0 kJ/mol.

According to previous results that were obtained for the half-sandwich type platinum group cations, the formation of dimeric species cannot be ruled out either. Since pH-potentiometry is not suitable to distinguish between the monomeric and dimeric species, the structures of [ML] and [M_2_L_2_] complexes were also computed in the [(η^6^-*p*-cym)Ru]^2+^–IdaP^3−^ system. In the dimeric species, in addition to a phosphonate–O,N chelate, the carboxylate group of IdaP^3−^ was assumed to coordinate to the second Ru ion and vice versa (Figure S4). Steric hindrance between the arene rings was not observed. The relative energy between the mono- and dimeric species was predicted as follows:

Δ*G* = *G*^tot^_aq_ (M_2_L_2_) − 2 x *G*^tot^_aq_ (ML)

and was found to be +1134.7 kJ/mol. This huge value suggests that the formation of dimeric species is not favorable in this system. The structure of the dimer is shown in Figure S4, while the Cartesian coordinates for the monomer and for the dimer can be found in Tables S3 and S4, respectively.

**Figure 3:**
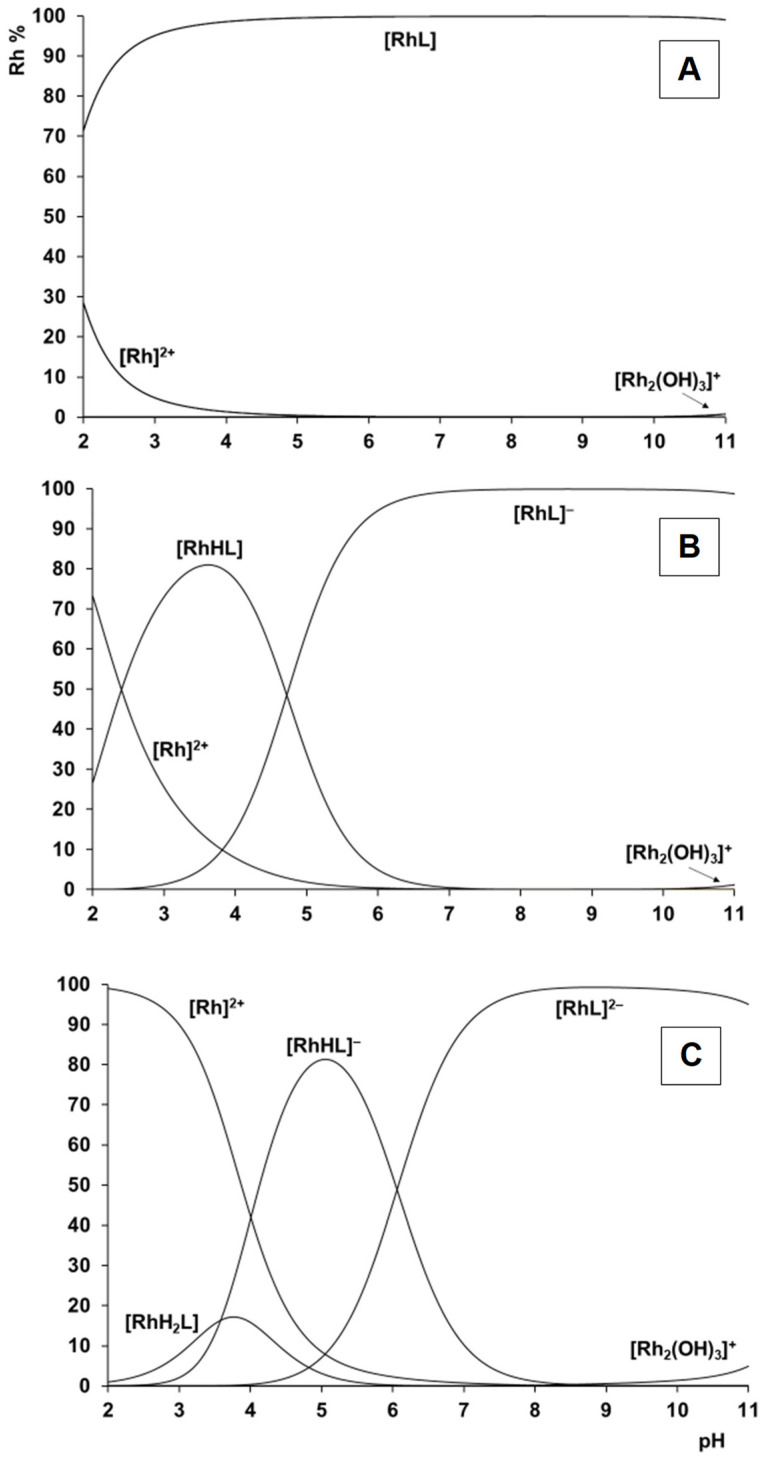
Concentration distribution curves calculated for the [(η^5^-Cp*)Rh]^2+^–Ida^2−^ (**A**), –IdaP^3−^ (**B**), and Ida2P^4−^ (**C**) systems at a 1:1 ratio (c_Ru_ = 3 mM, *I* = 0.20 M KCl). “Rh” stands for the [(η^5^-Cp*)Rh]^2+^ entity.

For Ida2P^4−^, again, due to the higher basicity of the phosphonate-O compared to the carboxylate-O, in addition to [MHL], an [MH_2_L]-type species can also be detected in a measurable concentration. The calculated p*K*_MH2L_ (2.79 for Ru and 3.58 for Rh) and p*K*_MHL_ (6.02 for Ru and 6.06 for Rh) values may support the deprotonation of the phosphonic functions in stepwise processes. The most plausible structures of these protonated species can be seen in Figure 2 as IV and V. As a representative example, the speciation diagrams using the stability constants in Table 2 are calculated and shown for the various rhodium systems in Figure 3. As shown in Figure 3, with Ida^2−^, a very stable single species is present over the 3–11 pH range. With IdaP^3−^, the amount of the uncomplexed metal ion is ~70% at a pH of 2.0, while this is 100% for Ida2P^4−^, which is in agreement with the basicity trends of the ligands. Nevertheless, in both of the latter systems at a pH of 7.4, the [ML] species are only present, and no hydrolysis of the metal ion is detectable up to a pH of 10.5.

In the organoosmium- and -iridium-containing systems, in general, slow complexation processes were detected. This was especially pronounced with Ida^2−^, where the titration curves could only be evaluated below pH ~ 6 with the osmium and below pH ~ 10 with iridium cation. One reason for this might be the much higher tendency of these metal ions to hydrolyze compared to the lighter congeners that were discussed before. As a result, only tentative stability constants with less accuracy could be determined (Table 2). Nevertheless, the models are identical to those that were found for the rhodium cation, with a comparable stability of the various species. However, as a representative example, the calculated speciation curves in Figure S5 revealed that, at pH 6.0 for the osmium–Ida^2−^ system, due to the discussed tendency of the metal ion to hydrolyze, ~50% of it can already be found in the form of the [{(η^6^-*p*-cym)Os}_2_(μ^2^-OH)_3_]^+^ hydroxido complex. The phosphonate ligands, again, can better prevent the 5*d* metal ions from hydrolysis compared to Ida^2−^, as indicated by the speciation diagrams that have been calculated for the [(η^6^-*p*-cym)Os]^2+^– and [(η^5^-Cp*)Ir]^2+^–Ida2P^4−^ systems (Figure S5). For the osmium- and –iridium-containing complexes, an identical binding mode of the ligands is plausible, as discussed previously.

The general comparison of the stability constants in Table 2 for the three different ligands considering the same metal ion reveals that the replacement of one carboxylate function by a phosphonate group with higher basicity results in the formation of more stable complexes. At the same time, the presence of the second phosphonate group in Ida2P^4−^ does not provide further stability increase; the log*β* values for the appropriate M–IdaP^3−^ and M–Ida2P^4−^ (M = [(η^6^-*p*-cym)Ru]^2+^, [(η^6^-*p*-cym)Os]^2+^, [(η^5^-Cp*)Rh]^2+^, or [(η^5^-Cp*)Ir]^2+^) complexes are comparable with each other. This lack of further increase in the stability may be interpreted in terms of the steric hindrance of the bulky phosphonate group resulting in the least favorable (5+5) joined chelates with the involvement of two phosphonate groups compared to that with one carboxylate and one phosphonate function.

**Figure 4:**
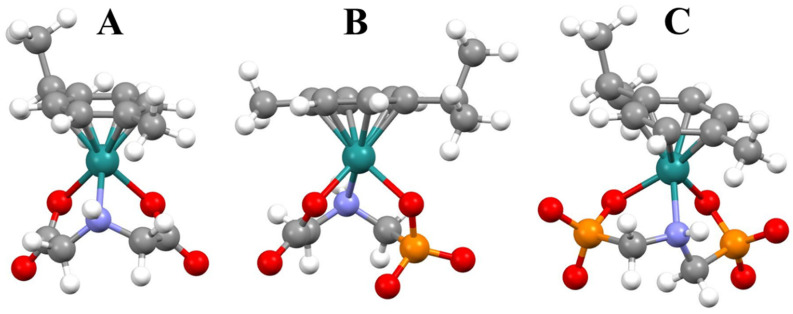
The DFT optimized structure of the [(η^6^-*p*-cym)Ru(Ida)] (**A**), [(η^6^-*p*-cym)Ru(IdaP)]^−^ (**B**), and [(η^6^-*p*-cym)Ru(Ida2P)]^2−^ (**C**).

In order to shed light on the above assumption, the effect of the incorporation of phosphonate group(s) was also studied through the DFT method, and the geometry of the [ML] complexes that formed between [(η^6^-*p*-cym)Ru]^2+^ and Ida^2−^, IdaP^3−^, or Ida2P^4−^ was also studied in detail. The optimized geometries are shown in Figure 4, the optimized bond lengths and angles for these complexes are summarized in Table 3, while the Cartesian coordinates are collected in Tables S3, S5, and S6, respectively.

As can be seen from the DFT calculations, all three of the ligands form stable complexes with the [(η^6^-*p*-cym)Ru]^2+^ cation in agreement with the speciation studies. In all cases, the three donor atoms of Ida^2−^ and its mono- and diphosphonate derivatives are involved in the coordination, and there is no steric repulsion, which hinders the formation of (O,N,O) chelated complexes. Noticeably, significant differences were found in the bond length values between the Ru ion and the arene ring for [(η^6^-*p*-cym)Ru(IdaP)]. This distance is longer than those that were obtained for the [(η^6^-*p*-cym)Ru(Ida)] and [(η^6^-*p*-cym)Ru(Ida2P)] complexes. This effect may explain the increased stability of [(η^6^-*p*-cym)Ru(IdaP)], which was obtained by pH-potentiometry, as the higher bond length between the Ru and the arene ring provides a higher intrinsic stability in a more relaxed structure.

In order to provide support for the above solution speciations and the assumed binding modes, pH-dependent NMR titrations at 1:1 and 1:2 metal ion to ligand ratios were also carried out in all of the systems. The NMR spectra that were acquired at a 1:2 ratio (not shown) did not indicate other signals than those that appeared at a 1:1 ratio, pointing out the exclusive formation of 1:1 complexes in agreement with pH-potentiometry.

**Figure 5:**
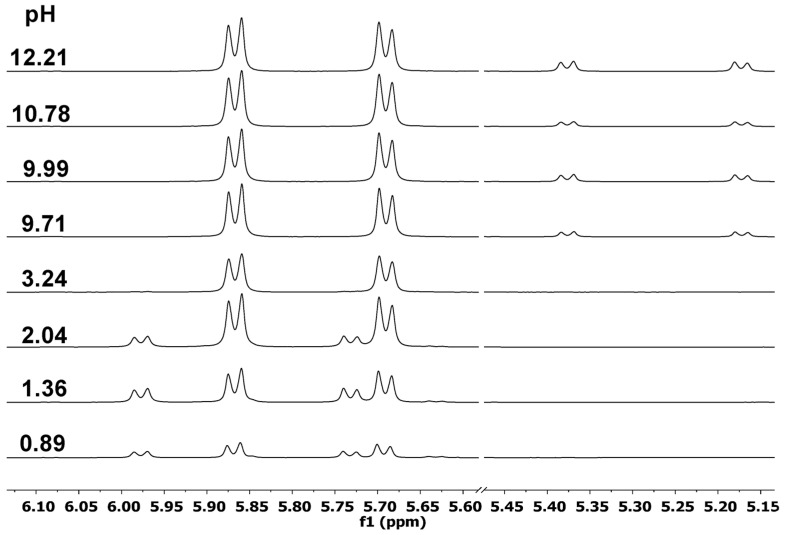
The pH-dependence of the ^1^H NMR signals belonging to the neighboring ring protons of [(η^6^-*p*-cym)Ru]^2+^ moiety registered with Ida^2−^ at a 1:1 ratio (c_Ru_ = 10 mM, *I* = 0.20 M KNO_3_).

In order to monitor the complex formation, the pH-dependence of ^1^H NMR spectra that was acquired in the ruthenium-Ida^2−^ system at a 1:1 metal ion to ligand ratio is shown in Figure 5. (Due to the slow exchange processes on the NMR time scale, each set of signals belonging to a given species can be seen separately. For clarity, only the low-field region of the spectra with the signals belonging to the neighboring ring protons of the coordinating *p*-cymene unit are shown in Figure 5).

At pH 0.89, in addition to the signals (5.73 and 5.98 ppm) of the free [(η^6^-*p*-cym)Ru]^2+^, a new pair of doublets (5.69 and 5.87 ppm) belonging to the complexed metal ion can be seen (Figure 5). Up to pH 3.2, the resonances of the complex—based on pH-potentiometry this is [ML]—are present only, and they remain the major signals in the whole measured pH range. Notably, the position of these signals does not change by increasing the pH, suggesting the presence of a single complex. This is in contrast to the speciation model that indicates the formation of both [MHL]- and [ML]-type species under acidic conditions with this ligand (Table 2). In order to shed light on this contradiction, time-dependent NMR measurements with 1:1 organoruthenium-Ida^2−^ samples at a pH of 2.17 and 2.98 were carried out. It was found that the formation of the complexed species is very slow (see Figure S7A), resulting in spectral changes even after three days. On the contrary, in the analogous rhodium system (Figure S7B), due to the fast complex formation, 10 min was enough to reach complete equilibrium. The very slow processes that were detected with [(η^6^-*p*-cym)Ru]^2+^ can explain the differences in the results that were obtained by potentiometry and NMR. Namely, no real equilibrium could be reached during the potentiometric titrations, with the usual set up of the titration parameters resulting in different titration curves upon re-acidification. Consequently, in the ruthenium-Ida^2−^ system, the evaluation of these non-equilibrium titration curves required the assumption of [MHL] (being a transient species based on NMR results) leaving less “pH-effect” for [ML], thus resulting in a lower-than-real stability constant for the latter complex. Since the rate of complex formation with the O donor ligands for the ruthenium cations is fast in general [34], the coordination of the imino-N to the ruthenium cation is most likely responsible for the slow processes. Slow exchange processes of N-coordinated ligands are also well documented in the literature [36].

**Figure 6:**
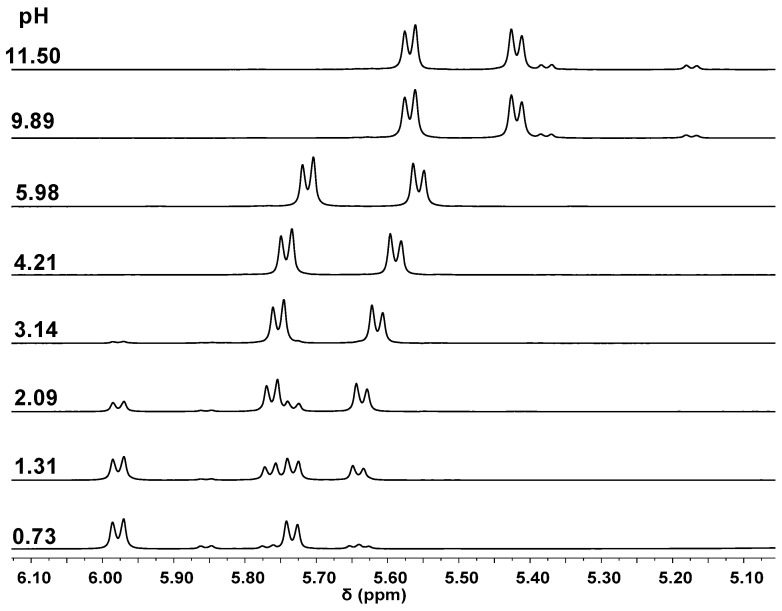
The pH-dependence of the ^1^H NMR signals belonging to the neighboring ring protons of [(η^6^-*p*-cym)Ru]^2+^ moiety registered with Ida2P^4−^ at a 1:1 ratio (c_Ru_ = 10 mM, *I* = 0.20 M KNO_3_).

For the [(η^6^-*p*-cym)Ru]^2+^–Ida2P^4−^ system (Figure 6), which also contains a symmetrical ligand (as above with Ida^2−^), the pH-dependence of the NMR spectra reveal that the free [(η^6^-*p*-cym)Ru]^2+^ (5.73 and 5.98 ppm) above pH ~ 3.5 is not detectable in a measurable concentration anymore, while a new set of signals belonging to the complexed species are observable as low as pH 1.3. Upon increasing the pH, the continuous upfield shift of them is in line with the stepwise deprotonation processes of [MH_2_L] and [MHL], resulting in the formation [ML] as the final complex. The hydrolysis of the metal ion that was indicated at 5.18 and 5.38 ppm (similarly to the data for the Ida^2−^ system, Figure 5) does not exceed 5%, even at pH 11.5.

**Figure 7:**
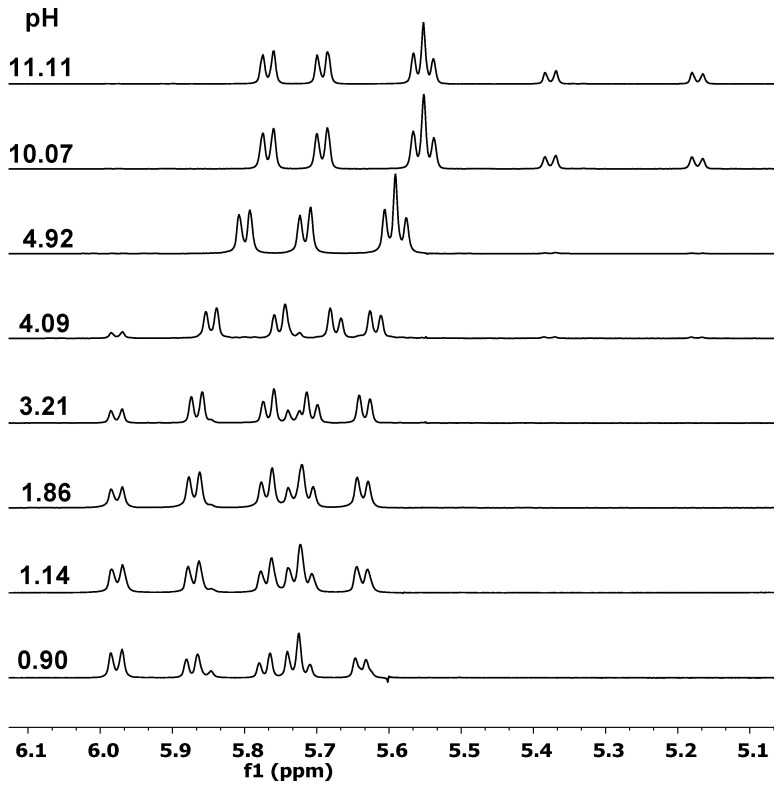
The pH-dependence of the ^1^H NMR signals belonging to the neighboring ring protons of [(η^6^-*p*-cym)Ru]^2+^ moiety registered with IdaP^3−^ at a 1:1 ratio (c_Ru_ = 10 mM, *I* = 0.20 M KNO_3_).

With IdaP^3−^ (Figure 7), the formation of stable complexes and the continuous shift of the resonances of the complexed species indicate similar processes as before. Unlike the previous systems with the symmetrical ligands, for the ruthenium–IdaP^3−^ 1:1 samples, four doublets belonging to the ring hydrogens of the hexahapto-bound *p*-cymene ligand are detected. This is due to the asymmetrical nature of the phosphonate ligand; therefore, upon tridentate (O,N,O) coordination, these hydrogens become non-equivalent, while the central metal ion acts as a stereogenic center. As a result, the two diastereomers that are formed have altogether four signals for these *p*-cym hydrogens. Minor hydrolysis of the metal ion is noticeable again above pH 10.

**Figure 8:**
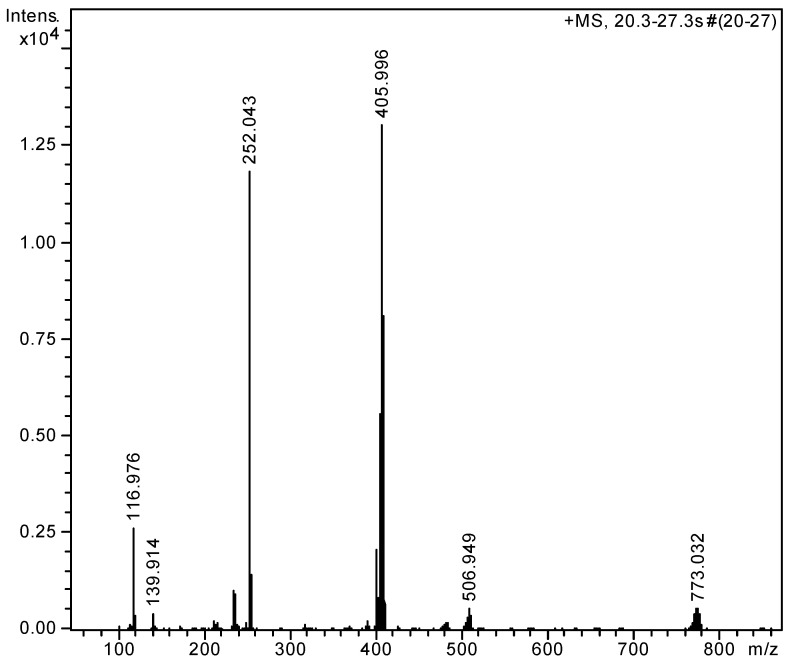
ESI-MS spectrum of an aqueous sample of the [(η^6^-*p*-cym)Ru]^2+^-Ida^2−^ system at a 1:1 ratio, pH = 9.0 (c_Ru_ = 0.1 mM).

**Figure 9:**
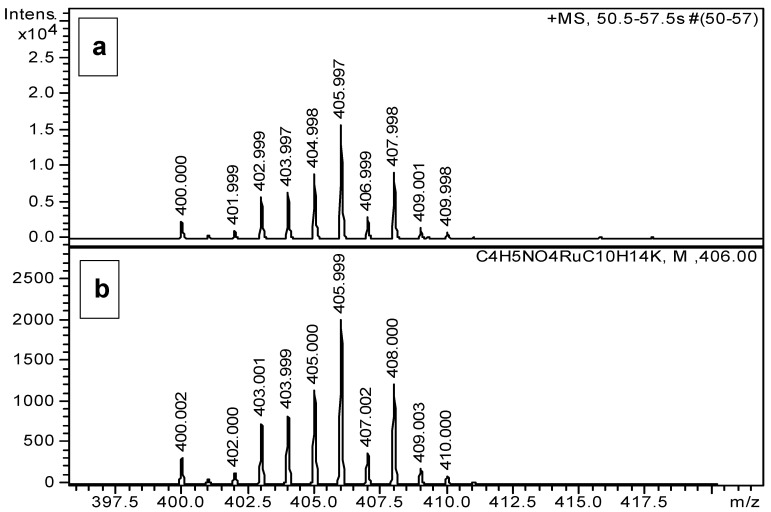
Measured (**a**) and calculated (**b**) isotope pattern of [(η^6^-*p*-cym)Ru(Ida)]+K^+^ ion.

Although ESI-TOF-MS may generate species that were not present in the aqueous sample, these measurements at different pH values in the positive mode have provided further valuable proof in our study for the existence of the above detailed complexes with the three ligands (Table S7). As can be seen in Table S7, all of the 1:1 species with a different protonation degree for the phosphonate ligands could be detected, and the measured values show excellent agreement with the corresponding simulated m/z values. For the organoosmium cation, an oxo-hydroxido complex with {[(η^6^-*p*-cym)Os]_2_(O)OH}^+^ stoichiometry could also be detected, which is formed under MS conditions only, in agreement with previous observations [37]. In all of these cases, the measured and calculated isotope patterns also showed very good agreement. As a representative example, Figure 8 exhibits the registered MS spectrum of the ruthenium-Ida^2−^ 1:1 system at pH 9.0, while in Figure 9, the measured and the simulated isotope distribution of the [(η^6^-*p*-cym)Ru(Ida)]+K^+^ cation is presented.

## 3. Materials and Methods

### 3.1. Materials

H_2_Ida was purchased from BDH, and H_3_IdaP, H_4_Ida2P, and KOH were delivered by Aldrich (St. Louis, MO, USA). KNO_3,_ HCl, and HNO_3_ were obtained from Molar Chemicals (Halásztelek, Hungary), KCl from Merck (Darmstadt, Germany), and AgNO_3_ from Reanal (Budapest, Hungary). NaOD was purchased from Cambridge Isotope Laboratories (Andover, MA, USA). The [(η^5^-Cp*)RhCl_2_]_2_ (99%) and [(η^5^-Cp*)IrCl_2_]_2_ (98%) were delivered by Strem Chemicals (Newburyport, MA, USA), while [(η^6^-*p*-cym)RuCl_2_]_2_ and [(η^6^-*p*-cym)OsCl_2_]_2_ were synthesized and purified according to previously published methods [38], using RuCl_3_·xH_2_O (Merck), α-terpinene (Acros, Geel, Belgium), and OsO_4_ (W. C. Heraeus GmbH, Hanau, Germany). The [(η^6^/η^5^-arene/arenyl)M(H_2_O)_3_](NO_3_)_2_ stock solutions were obtained from the appropriate dimeric precursors, with the removal of chloride ions using equivalent amounts of silver nitrate.

### 3.2. Solution Studies

For the solution studies, doubly deionized and ultra-filtered water was obtained from a Milli-Q RG (Millipore, Burlington, MA, USA) water purification system. The pH-potentiometric measurements were carried out at a constant ionic strength of 0.20 M KCl at 25.0 °C. Carbonate-free KOH solutions of known concentrations (ca. 0.2 M) were used as the titrant. HCl and HNO_3_ stock solutions were prepared from concentrated HCl or HNO_3_, respectively, and their concentrations were determined by potentiometric titrations using the Gran’s method [39]. A Mettler Toledo DL50 (Nänikon, Switzerland) titrator, which was equipped with a DG114-SC combined glass electrode, was used for the pH-potentiometric measurements. The electrode systems were calibrated according to Irving et al. [40], and the pH-metric readings could, therefore, be converted into a hydrogen ion concentration. The water ionization constant, p*K*_w_, was 13.76 ± 0.01 under the conditions employed. The initial volume of the samples was 15.00 mL. The metal ion concentrations varied in the range of 1.3–2.8 mM. The samples were, in all cases, completely deoxygenated by bubbling purified argon ca. for 20 min before the measurements. The titrations were performed in the pH range of 2.0–11.0 in an equilibrium-controlled mode, during which the pH equilibrium was assumed to be reached if a change in the measured potential was less than 0.1 mV within 90 s. The minimum waiting time was 2 min, while the maximum was up to 45 min, due to the slow equilibrium processes. The protonation constants of the ligands and the overall stability constants of the complexes (β_p,q,r_ = [M_p_H_q_L_r_]/[M]^p^[H]^q^[L]^r^, where “M” stands for [(η^6^-*p*-cym)Ru(H_2_O)_3_]^2+^, [(η^6^-*p*-cym)Os(H_2_O)_3_]^2+^, [(η^5^-Cp*)Rh(H_2_O)_3_]^2+^, or [(η^5^-Cp*)Ir(H_2_O)_3_]^2+^, and “L” represents the completely deprotonated form of the ligands) were calculated with the aid of the SUPERQUAD [41] and PSEQUAD [42] computer programs, respectively. During the calculations, the hydrolysis of the metal ions was taken into consideration. The stability constants of the various hydroxido complexes in the chloride- or nitrate-containing medium involved in the equilibrium models were taken from the literature [33,37,43].

For ^1^H NMR titrations, spectra were recorded on a Bruker AM360 NMR instrument at I = 0.20 M KCl or KNO_3_ and 20 °C. Titrations were carried out in D_2_O (99.8%) at c_M_ = 4.47–10.00 mM in order to register the pH* or time dependence of the chemical shifts. The pH* was set up with NaOD or DNO_3_ in D_2_O. Individual samples were equilibrated for 60 min before the measurements were taken, except for the time dependence studies. The pH* values (direct pH meter readings in a D_2_O solution of a pH meter calibrated in H_2_O according to Irving et al. [40]) measurable at an ionic strength of 0.20 M were converted into pH values using the following equation: pH = 0.936pH* + 0.412 [44]. The NMR spectra were analyzed using the MestreNova program (version 14.1.1).

ESI-TOF MS analysis in positive mode was carried out with a Bruker maXis II UHR ESI-TOF MS instrument. The measurements were performed in water at 0.5 mM metal ion concentration at different pH values. The temperature of the drying gas (N_2_) was 100 °C. The pressure of the nebulizing gas (N_2_) was 30 psi. The voltages that were applied at the capillary entrance, capillary exit, and the first and the second skimmers were −4500, 120, 40, and 30 V, respectively. The spectra were accumulated and recorded with a digitalizer at a sampling rate of 2 GHz.

### 3.3. DFT Calculations

The geometry optimization of the half-sandwich ruthenium(II) complexes was computed through Gaussian 16 (AM64L-G16 RevB.01) [45] at a DFT level of theory by the use of the hybrid meta-GGA wB97XD functional [46], which contained an empirical dispersion term to describe medium and large interatomic distances [47]. The relativistic small-core ECPs SDD [48] and LANL2DZ [49] were employed on the ruthenium, and the *def2*-TZVP basis set was used for H, C, N, O, and P atoms. All calculations accounted for solvent effect using the SMD [50], which is based on the quantum mechanical charge density of a solute molecule interacting with a continuum description of the solvent. This model, with the corresponding functional and basis set, has already demonstrated its high degree of accuracy in the prediction of the geometry of ruthenium complexes [51]. Single-point frequency calculations were carried out with the same functional and basis sets for the ground state geometries, which represented true minima on the potential energy surface (no imaginary frequencies were found).

## 4. Conclusions

The analysis and the comparison of the potentiometric, NMR, MS, and DFT information has allowed us to draw the following major conclusions: (i) For Ida^2−^, similarly to the organoruthenium system, with the organorhodium cation, a 1:1 species with high stability and with tridentate coordination of the ligand is present over a wide pH range and is capable of hindering the hydrolysis of the metal ion completely, even at pH 11.0. With the heavier counterparts, Os(II) and Ir(III), although complexes with identical stoichiometry and binding mode are formed in the acidic pH range, the presence of [M_2_(μ^2^-OH)_3_]^+^ (M = [(η^6^-*p*-cym)Os]^2+^ or [(η^5^-Cp*)Ir]^2+^) as major species that were detected above pH 3.5 (Os) and 6.0 (Ir) clearly indicates that the tridentate ligand is not able to prevent these metal ions from intensive hydrolysis. This trend is in agreement with the tendency for hydrolysis of the various metal aquo species [37]. (ii) The replacement of one carboxylate by a phosphonate group in IdaP^3−^ results in a significant increase in the stability of the complexes with tridentate coordination. This is clearly reflected in the lack of, or almost negligible extent of, hydrolysis, even for the samples with Os(II) and Ir(III). Notably, as was found with the organoruthenium cation, in the Os(II)-containing samples, the formation of two stereoisomers is clearly seen, which was due to the asymmetric structure of the tridentate ligand. (With IdaP^3−^ in the Rh(III) and Ir(III) systems, diastereomers are also present, however, due to the very small change in the chemical shift value belonging to the protons of the Cp* arenyl ligand, this is hardly seen in most of the cases in the NMR spectra.) (iii) With Ida2P^4−^, there is no further increase in the stability of the complexes that are present in the solution, although this ligand contains two phosphonate groups with a higher basicity than carboxylate. Based on DFT, this can be interpreted in terms of a higher intrinsic stability in a more relaxed structure for the (O,N,O)-coordinated [ML] with IdaP^3−^. (iv) For all of the metal ions with IdaP^3−^ and Ida2P^4−^, the continuous upfield shift of the NMR signals belonging to the complexes indicate the deprotonation of the [MH_2_L] and [MHL] species with increasing pH. These deprotonations most likely occur at the non-coordinating -OH group(s) of the ligands without affecting the (O,N,O) binding mode, which is also supported by the calculations.

## Figures and Tables

**Table 1:** Stepwise protonation constants (log*K_i_*) of the ligands at 25.0 °C, *I* = 0.20 M KCl *.

	Ida^2−^	IdaP^3−^	Ida2P^4−^
log*K*_1_	9.25 (1)	10.01 (1)	10.81 (1)
log*K*_2_	2.57 (2)	5.40 (1)	5.98 (1)
log*K*_3_	1.67 (5)	2.21 (1)	4.92 (1)
log*K*_4_	–	<1	<1
log*K*_5_	–	–	<1

* 3σ standard deviations are in parentheses.

**Table 2:** Overall stability constants (log*β*) of the [(η^6^-*p*-cym)Ru]^2+^, [(η^5^-Cp*)Rh]^2+^, [(η^6^-*p*-cym)Os]^2+^ and [(η^5^-Cp*)Ir]^2+^ complexes of Ida^2−^ and its phosphonate derivatives, IdaP^3−^ and Ida2P^4−^ together with some calculation parameters and derived p*K* values (*T* = 25.0 °C, *I* = 0.20 M KCl) *.

		[(η^6^-*p*-cym)Ru]^2+^	[(η^5^-Cp*)Rh]^2+^	[(η^6^-*p*-cym)Os]^2+^	[(η^5^-Cp*)Ir]^2+^
Ida^2−^	log*β* [MHL]^+^	12.81 (5)	—	—	—
	log*β* [ML]	10.26 (2)	11.53 (4)	10.1 (1)	11.9 (1)
	Fitting parameter ^#^	0.0117	0.00420	0.01505	0.00887
	Number of data points fitted	141	146	184	123
	p*K*_[MHL]_	2.55	—	—	—
IdaP^3−^	log*β* [MHL]	17.44 (1)	16.05 (1)	18.5 (1)	17.9 (1)
	log*β* [ML]^−^	13.00 (1)	11.33 (2)	14.9 (1)	12.6 (1)
	Fitting parameter ^#^	0.00388	0.00787	0.01645	0.00868
	Number of data points fitted	154	207	156	225
	p*K*_[MHL]_	4.44	4.72	3.6	5.3
Ida2P^4−^	log*β* [MH_2_L]	21.92 (1)	20.23 (1)	21.7 (1)	23.0 (1)
	log*β* [MHL]^−^	19.13 (1)	16.65 (4)	19.4 (1)	18.8 (1)
	log*β* [ML]^2−^	13.11 (1)	10.59 (7)	13.8 (1)	13.0 (1)
	Fitting parameter ^#^	0.00526	0.00306	0.00797	0.00853
	Number of data points fitted	220	164	284	249
	p*K*_[MH2L]_	2.79	3.58	2.3	4.2
	p*K*_[MHL]_	6.02	6.06	5.6	5.8

* 3σ standard deviations are in parentheses; ^#^ Fitting parameter is the average difference between the calculated and experimental titration curves expressed in mL of the titrant.

**Table 3:** Optimized bond lengths (Å) and angles (°) for the [(η^6^-*p*-cym)Ru(Ida)], [(η^6^-*p*-cym)Ru(IdaP)]^−^, and [(η^6^-*p*-cym)Ru(Ida2P)]^2−^ complexes.

Bond Lengths (Å)/Angles (°)	Ida	IdaP	Ida2P
Ru–Ct ^a^	1.623	1.730	1.662
Ru–OOC (1)	2.127	2.137	
Ru–OOC (2)	2.149		
Ru–OPOO (1)		2.159	2.155
Ru–OPOO (2)			2.149
Ru–NH	2.156	2.174	2.184
(1) COO–Ru–NH	79.0	78.1	
HN–Ru–OOC (2)	77.2		
(1) OOPO–Ru–NH		82.8	77.8
HN–Ru–OPOO (2)			82.6

^a^ Ct indicates the centroid of the arene ring.

## Data Availability

The data presented in this study are available on request from the corresponding author.

## Supplementary Materials

The following supporting information can be downloaded at: https://www.mdpi.com/article/10.3390/molecules28031477/s1. Figure S1. pH-potentiometric titration curves with Ida^2−^ (A), IdaP^3−^ (B) and Ida2P^4−^ (C) for the H^+^ – ligand system (a), and [(η^6^-*p*-cym)Ru]^2+^ – ligand systems; Figure S2. pH-potentiometric titration curves with Ida^2−^ (A), IdaP^3−^ (B) and Ida2P^4−^ (C) for the H^+^ – ligand system (a), and [(η^6^-*p*-cym)Os]^2+^ – ligand systems; Figure S3. Optimized structures of the two coordination isomers of [(η^6^-*p*-cym)Ru(HIdaP)] A and B; Figure S4. Optimized structure of the [M_2_L_2_] type dimer assumed in the [(η^6^-*p*-cym)Ru]^2+^–IdaP^3−^ system; Figures S5 and S6. Concentration distribution curves; Figure S7. Time dependence of the high field region of ^1^H NMR spectra recorded in the [(η^6^-*p*-cym)Ru]^2+^–Ida^2−^ (A) and [(η^5^-Cp*)Rh]^2+^–Ida^2−^ (B) 1:1 systems; Tables S1–S6. Cartesian coordinates and IR spectra of the various [(η^6^-*p*-cym)Ru(II)] complexes; Table S7. ESI-MS measured and calculated m/z values of the complexed species.
