# Supplementary material for: Interaction between [(η6-p-cym)M(H2O)3]2+ (MII = Ru, Os) or [(η5-Cp*)M(H2O)3]2+ (MIII = Rh, Ir) and Phosphonate Derivatives of Iminodiacetic Acid: A Solution Equilibrium and DFT Study
Source: Molecules. 2023 Feb 3;28(3):1477. doi: 10.3390/molecules28031477 (PMC9918899; doi:10.3390/molecules28031477)
Supplement: Supplementary file 1 [file molecules-28-01477-s001.zip › molecules-2111507-supplementary.pdf]

molecules

Supplementary Materials

Interaction between $[(\eta^6\text{-}p\text{-cym})M(H_2O)_3]^{2+}$ (M^{II} = Ru, Os) or $[(\eta^5\text{-Cp}^*)M(H_2O)_3]^{2+}$ (M^{III} = Rh, Ir) and Phosphonate Derivatives of Iminodiacetic Acid: A Solution Equilibrium and DFT Study

Linda Bíró, Botond Tóth, Norbert Lihi, Etelka Farkas and Péter Buglyó *

Department of Inorganic & Analytical Chemistry, Faculty of Science and Technology, University of Debrecen, H-4032 Debrecen, Egyetem tér 1, Hungary; linda.biro@science.unideb.hu (L.B.); toth.botond94@gmail.com (B.T.); lihi.norbert@science.unideb.hu (N.L.); efarkas@science.unideb.hu (E.F.)

* Correspondence: buglyo@science.unideb.hu

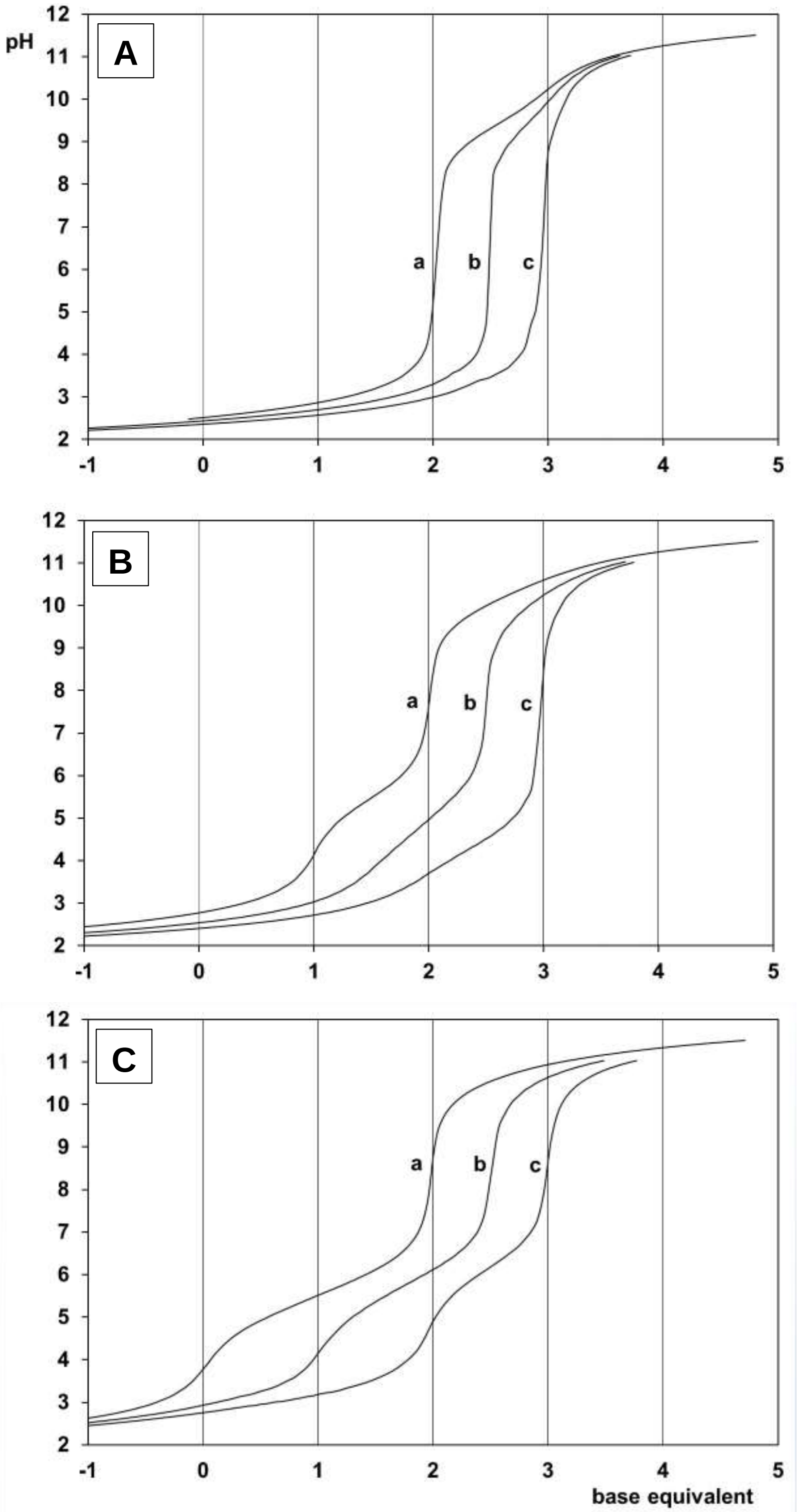

Figure S1. pH-potentiometric titration curves with Ida^{2-} (A), $IdaP^{3-}$ (B) and $Ida2P^{4-}$ (C) for the H^+ – ligand system (a), and $[(\eta^6\text{-}p\text{-cym})Ru]^{2+}$ – ligand systems at 1:2 (b) and 1:1 (c) ratios. Negative base equivalent refers to an excess of acid in the sample. I = 0.20 M KCl, t = 25.0 °C.

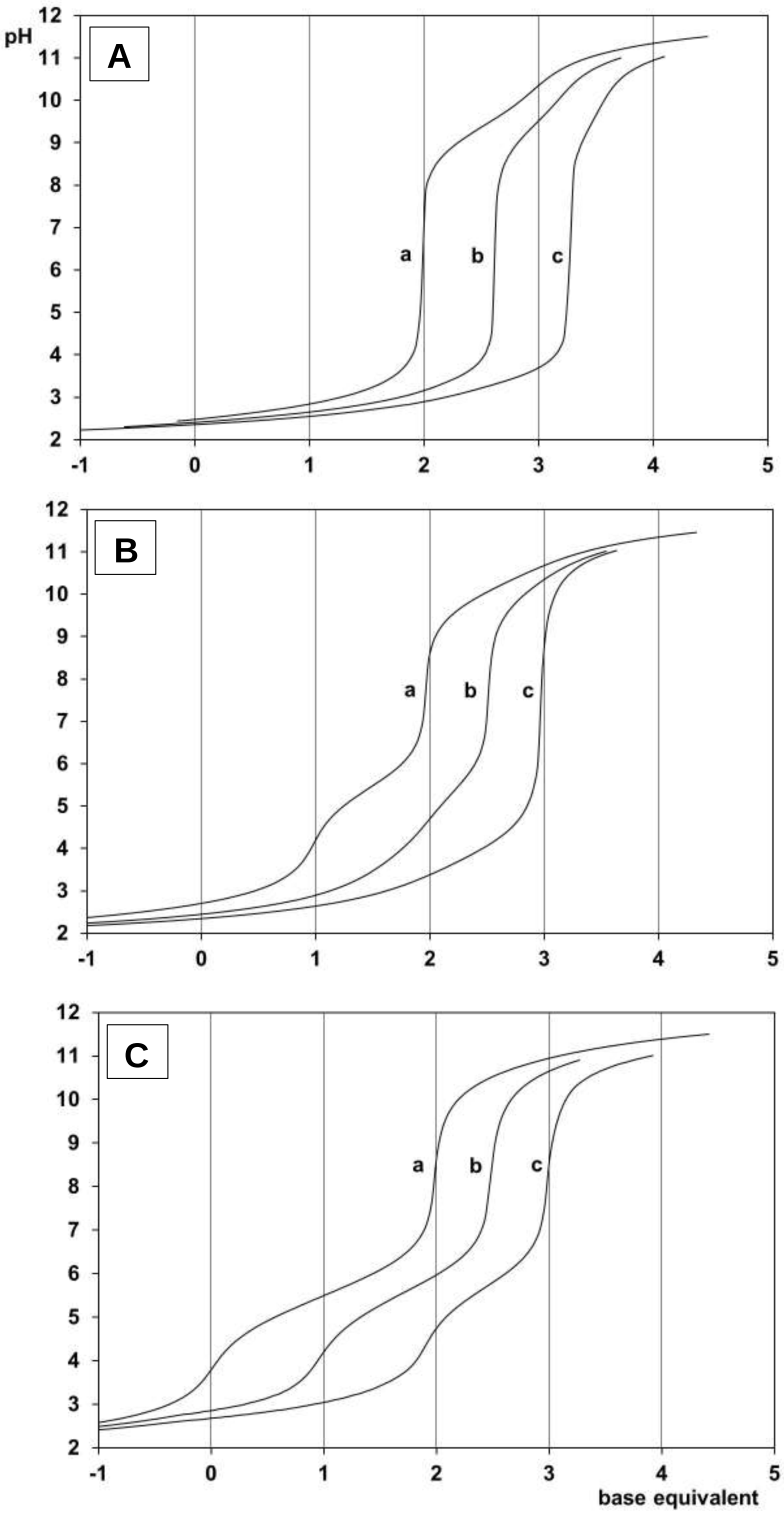

Figure S2. pH-potentiometric titration curves with Ida^{2-} (A), $IdaP^{3-}$ (B) and $Ida2P^{4-}$ (C) for the H^{+} – ligand system (a), and $[(\eta^6\text{-}p\text{-cym})Os]^{2+}$ – ligand systems at 1:2 (b) and 1:1 (c) ratios. Negative base equivalent refers to an excess of acid in the sample. I = 0.20 M KCl, t = 25.0 °C.

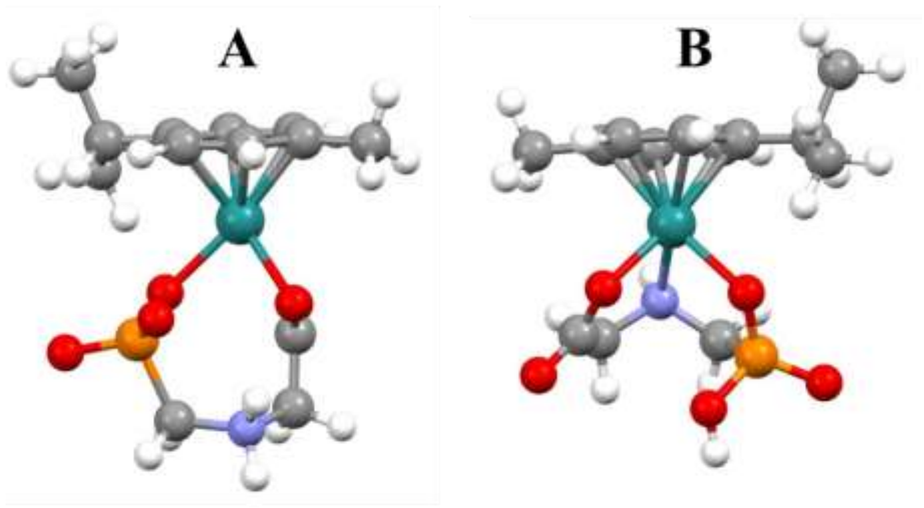

Figure S3. Optimized structures of the two coordination isomers of [(η^6-*p*-cym)Ru(HIdaP)] **A** and **B**.

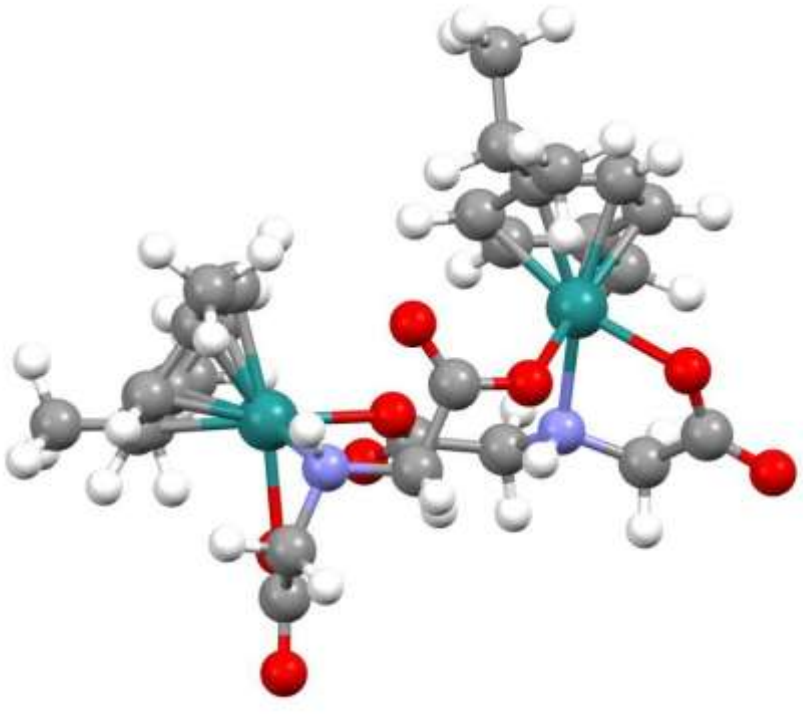

Figure S4. Optimized structure of the [M_2L_2] type dimer assumed in the [(η^6-*p*-cym)Ru]$^{2+}$–IdaP^{3-} system.

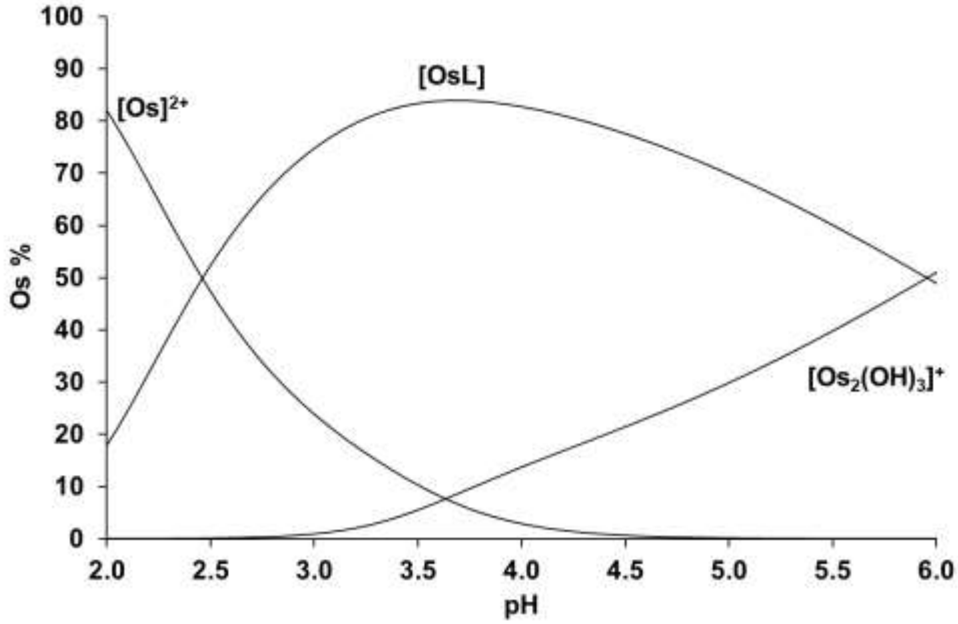

Figure S5. Concentration distribution curves calculated for the [(η[6]-*p*-cym)Os]$^{2+}$ – Ida$^{2–}$ system at 1:1 ratio (c = 3 mM, I = 0.20 M KCl). [Os]$^{2+}$ stands for the [(η[6]-*p*-cym)Os]$^{2+}$ entity.

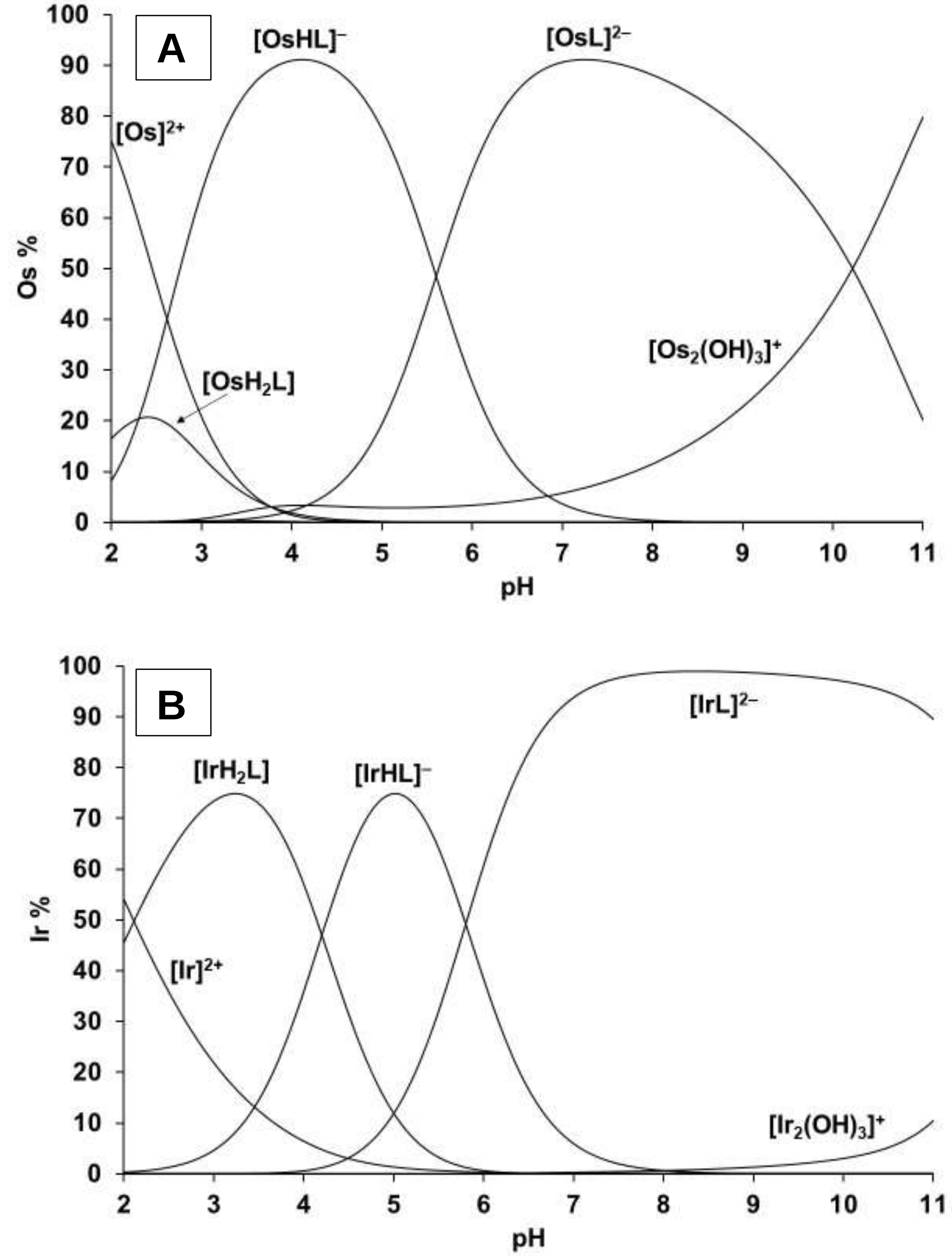

Figure S6. Concentration distribution curves calculated for the $[(\eta^6\text{-}p\text{-cym})Os]^{2+}$ – Ida2P^{4-} (A) and $[(\eta^5\text{-}Cp^*)Ir]^{2+}$ – Ida2P^{4-} (B) system at 1:1 ratio (c_{Os} = 3 mM, c_{Ir} = 0.8 mM, I = 0.20 M KCl). $[Os]^{2+}$ and $[Ir]^{2+}$ stand for the $[(\eta^6\text{-}p\text{-cym})Os]^{2+}$ and $[(\eta^5\text{-}Cp^*)Ir]^{2+}$ entities, respectively.

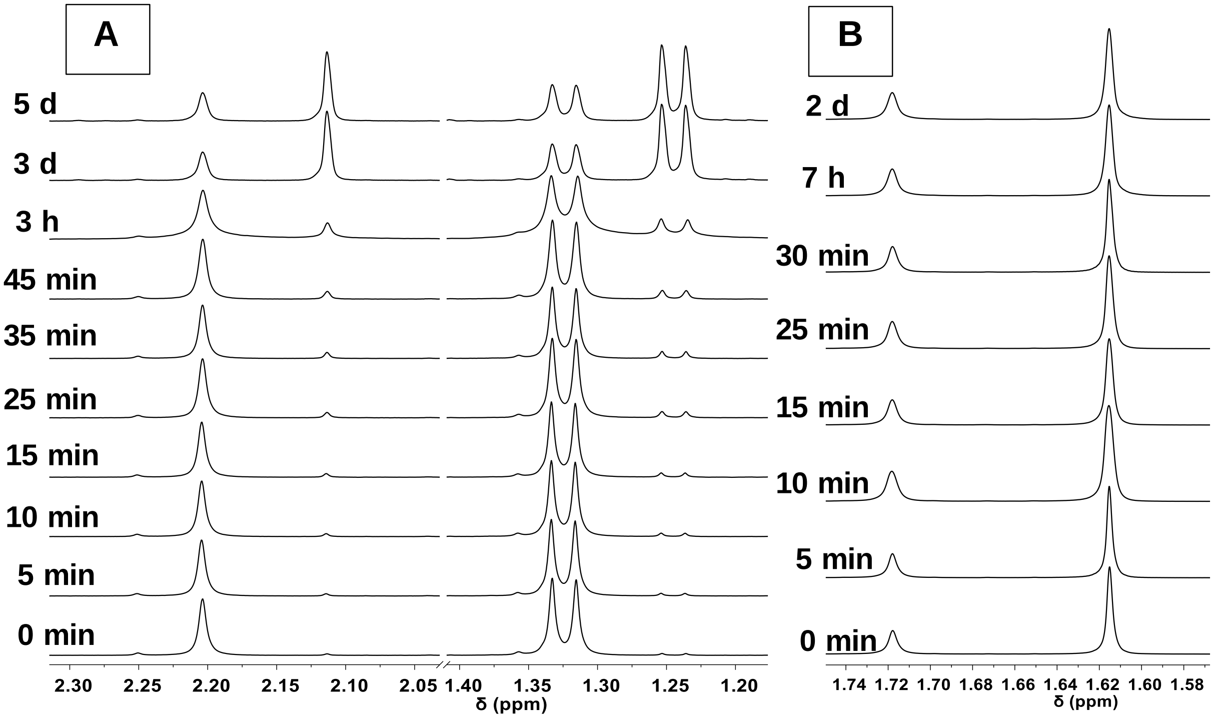

Figure S7. Time dependence of the high field region of ^{1}H NMR spectra recorded in the [(η^{6}-*p*-cym)Ru]$^{2+}$ – Ida^{2-} (A) and [(η^{5}-Cp*)Rh]$^{2+}$ – Ida^{2-} (B) 1:1 systems in D_2O (c_M = 5 mM, I = 0.20 M KCl). pH = 2.17.

Table S1. Cartesian coordinates and IR spectrum of [(η^6-*p*-cym)Ru(HIdaP)] complex (Isomer A).

Electronic Energy (Eh)	-1373.8499679000001
Sum of electronic and zero-point Energies (Eh)	-1373.517329
Sum of electronic and thermal Energies (Eh)	-1373.495354
Sum of electronic and enthalpy Energies (Eh)	-1373.494409
Sum of electronic and thermal Free Energies (Eh)	-1373.56835
Number of Imaginary Frequencies	0

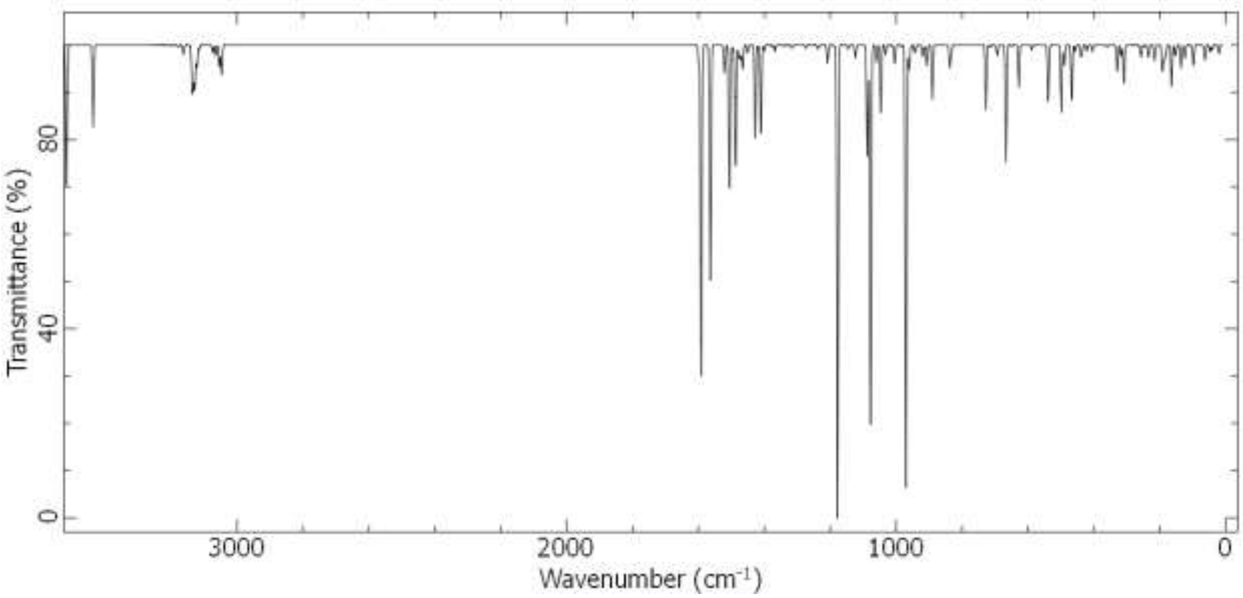

Molecular Geometry in Cartesian Coordinates

Ru	0.479221	-0.588885	-0.122054
C	1.833934	0.249445	1.328976
C	1.612153	-1.100787	1.645343
C	1.835715	-2.129555	0.688021
C	2.274029	-1.744144	-0.587531
C	2.492419	-0.378254	-0.912390
C	2.289469	0.637070	0.038352
H	1.567940	1.012558	2.047827
H	1.167925	-1.354242	2.598677
H	2.350084	-2.488446	-1.368607
H	2.734961	-0.122800	-1.933705
C	2.467135	2.101374	-0.269203
H	1.728595	2.634207	0.334751
C	2.234048	2.457399	-1.730904
H	1.265345	2.094937	-2.079107
H	2.254342	3.541775	-1.847296
H	3.012003	2.042307	-2.375388
C	3.862578	2.524815	0.194243
H	3.999867	3.595987	0.038532
H	4.011562	2.310922	1.254299
H	4.631274	1.994897	-0.373759

C	1.521193	-3.553287	1.010006
H	2.382366	-4.014786	1.498355
H	0.672091	-3.617747	1.689790
H	1.297830	-4.116087	0.104644
C	-1.813203	-1.422131	-0.931754
O	-0.978391	-1.308132	-1.853263
O	-1.456186	-1.617230	0.263500
C	-3.288317	-1.227843	-1.183031
H	-3.790040	-2.194577	-1.176280
H	-3.469667	-0.726826	-2.129032
N	-3.864465	-0.403298	-0.087537
H	-4.877420	-0.508135	-0.117006
C	-3.535442	1.058955	-0.131501
H	-4.324169	1.560512	0.427254
H	-3.597831	1.376661	-1.170794
P	-1.932400	1.591335	0.627948
O	-2.034448	3.095488	0.577901
O	-1.877497	0.960679	1.999167
O	-0.807763	1.093340	-0.314434
H	-3.545520	-0.788638	0.806058

Table S2. Cartesian coordinates and IR spectrum of [(η^6-*p*-cym)Ru(HIdaP)] complex (Isomer B).

Electronic Energy (Eh)	-1373.87436764
Sum of electronic and zero-point Energies (Eh)	-1373.543176
Sum of electronic and thermal Energies (Eh)	-1373.521365
Sum of electronic and enthalpy Energies (Eh)	-1373.520421
Sum of electronic and thermal Free Energies (Eh)	-1373.593119
Number of Imaginary Frequencies	0

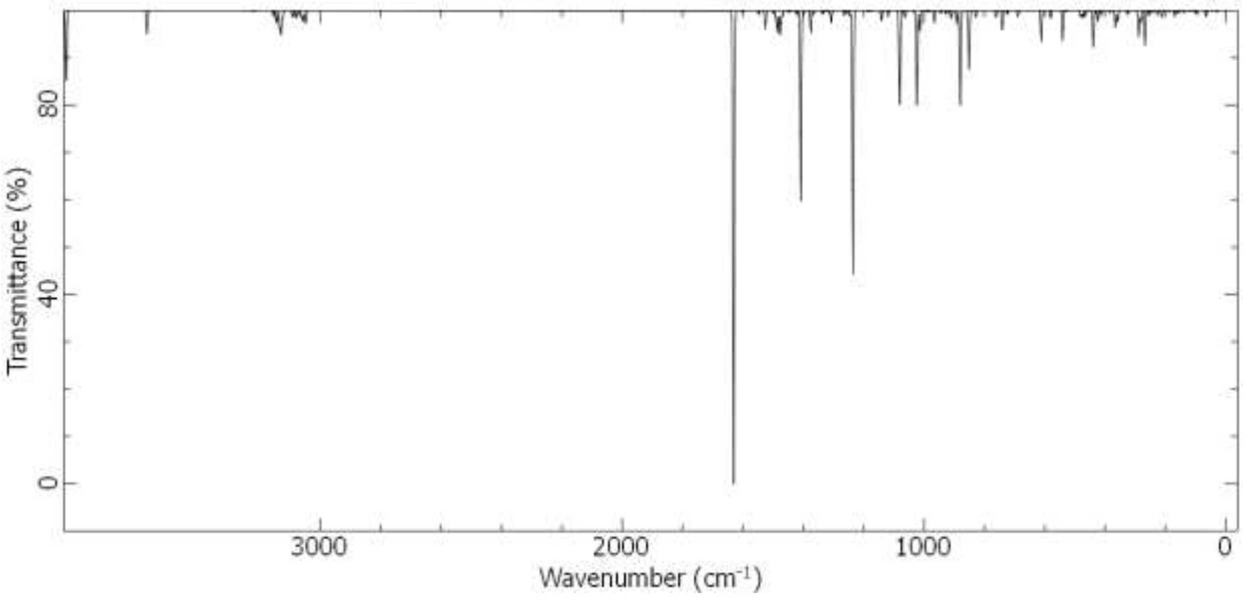

Molecular Geometry in Cartesian Coordinates

Ru	-0.212064	0.483883	-0.049217
C	-1.934138	0.392768	1.297494
C	-1.291465	1.650075	1.403676
C	-1.060492	2.464225	0.273077
C	-1.503119	1.969304	-0.982116
C	-2.135294	0.721690	-1.105573
C	-2.353841	-0.089063	0.041362
H	-2.037495	-0.232943	2.173042
H	-0.883848	1.953965	2.359643
H	-1.250196	2.520222	-1.878820
H	-2.388125	0.357321	-2.089520
C	-2.995444	-1.452167	-0.042732
H	-2.459681	-2.091763	0.663013
C	-2.930679	-2.092337	-1.422814
H	-1.910711	-2.127661	-1.806198
H	-3.307473	-3.114308	-1.363629
H	-3.552452	-1.553312	-2.141086
C	-4.446769	-1.326118	0.429770
H	-4.929050	-2.304762	0.413900
H	-4.504695	-0.931389	1.445366
H	-5.007157	-0.659654	-0.230632
C	-0.325555	3.761222	0.377892
H	-1.036179	4.587076	0.454583
H	0.310948	3.774032	1.262108
H	0.291619	3.925823	-0.505252
C	2.565126	1.205362	-0.743565
O	3.581729	1.545880	-1.354182
O	1.424319	1.042614	-1.293789
C	2.636908	1.023031	0.761104
H	2.636642	2.026025	1.191182
H	3.578396	0.543508	1.028430
N	1.488765	0.285823	1.315680
H	1.220615	0.736059	2.181284
C	1.749005	-1.133471	1.625089
H	0.989447	-1.462170	2.335557
H	2.730414	-1.268588	2.085421
P	1.552710	-2.175203	0.154065
O	1.514779	-3.615113	0.522543
O	2.818715	-1.848688	-0.783448
O	0.367667	-1.576601	-0.580188
H	3.624655	-2.292886	-0.497030

Table S3. Cartesian coordinates and the IR spectrum of [(η^6-*p*-cym)Ru(Ida)] complex.

Electronic Energy (Eh)	-994.6751368219999
Sum of electronic and zero-point Energies (Eh)	-994.355145
Sum of electronic and thermal Energies (Eh)	-994.335249
Sum of electronic and enthalpy Energies (Eh)	-994.334305
Sum of electronic and thermal Free Energies (Eh)	-994.403536
Number of Imaginary Frequencies	0

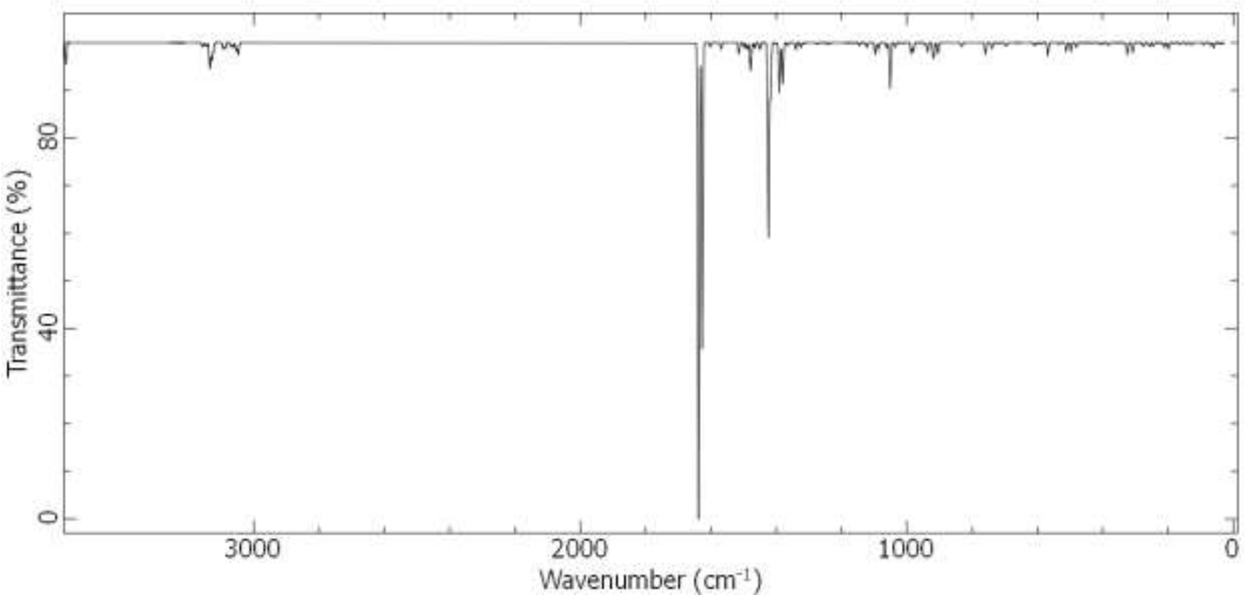

Molecular Geometry in Cartesian Coordinates

Ru	-0.020231	0.200561	-0.041185
C	1.724562	0.433190	-1.337523
C	0.825829	1.476436	-1.584740
C	0.349742	2.306991	-0.526317
C	0.824322	2.040260	0.767077
C	1.753253	0.991789	1.020509
C	2.202775	0.167873	-0.018145
H	2.006598	-0.228058	-2.145573
H	0.415707	1.600303	-2.578515
H	0.414627	2.592059	1.602415
H	2.032087	0.780565	2.042361
C	3.128776	-1.001526	0.199932
H	2.852449	-1.754620	-0.542365
C	3.010296	-1.630131	1.581431
H	1.975967	-1.893304	1.810099
H	3.611265	-2.539748	1.619978
H	3.376763	-0.959444	2.361602
C	4.561934	-0.551053	-0.089720
H	5.245612	-1.397255	-0.004647
H	4.653156	-0.137744	-1.095987
H	4.873745	0.214465	0.625065
C	-0.658630	3.377838	-0.790409
H	-0.152829	4.278198	-1.146143
H	-1.365206	3.063927	-1.559547
H	-1.207372	3.627527	0.116707
C	-2.550088	0.104577	1.462371
O	-3.407732	0.044758	2.347813
O	-1.307479	-0.120832	1.649653
C	-2.951094	0.520929	0.060054
H	-2.970093	1.612666	0.051891
H	-3.953529	0.160309	-0.171489
N	-1.973929	0.078800	-0.945872
H	-2.008904	0.727228	-1.722487

C	-2.197731	-1.283030	-1.462087
H	-1.969400	-1.286805	-2.529038
H	-3.239392	-1.580671	-1.343503
C	-1.292309	-2.327184	-0.832323
O	-0.247312	-1.905188	-0.232141
O	-1.571652	-3.520943	-0.972607

Table S4. Cartesian coordinates and the IR spectrum of the ((η[6]-*p*-cym)Ru)$_2$(Ida)$_2$] complex.

Electronic Energy (Eh)	-1989.82018446
Sum of electronic and zero-point Energies (Eh)	-1989.17487600
Sum of electronic and thermal Energies (Eh)	-1989.13453002
Sum of electronic and enthalpy Energies (Eh)	-1989.13358581
Sum of electronic and thermal Free Energies (Eh)	-1989.23925740
Number of Imaginary Frequencies	0

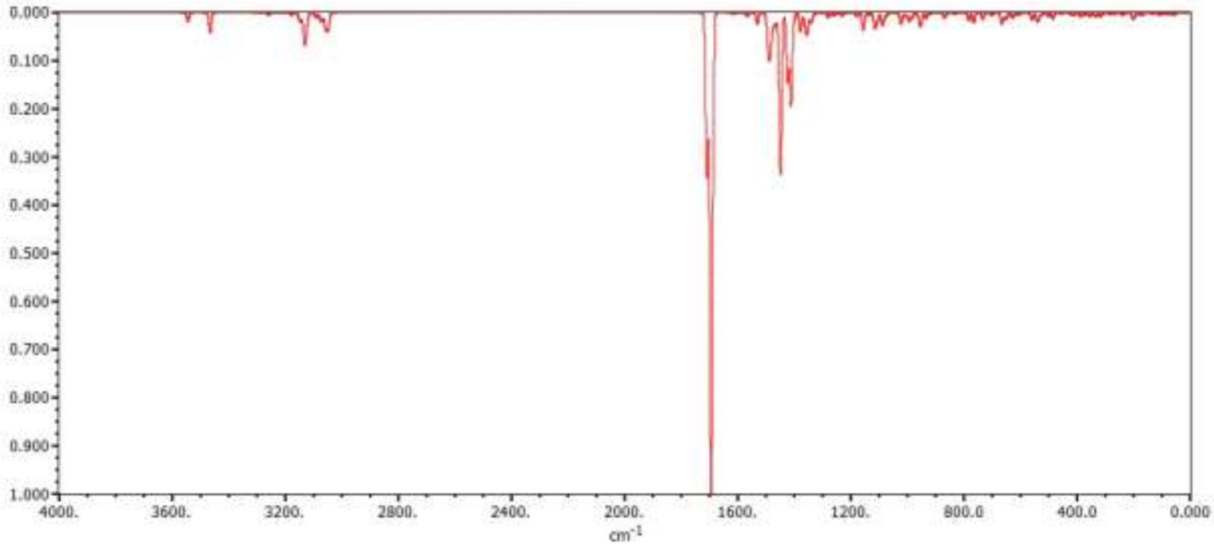

Molecular Geometry in Cartesian Coordinates

Ru	2.54817	0.05796	0.18195
C	4.54873	0.82156	0.26772
C	3.66676	1.90721	0.15324
C	2.53781	2.05021	1.00335
C	2.41488	1.11698	2.04977
C	3.30217	0.02884	2.20109
C	4.37276	-0.13462	1.30350
H	5.33320	0.67899	-0.46390
H	3.75235	2.55520	-0.71178
H	1.52361	1.13532	2.66101
H	3.08696	-0.72232	2.94203
C	5.29191	-1.32429	1.36118
H	5.53670	-1.57828	0.32581

C	4.66312	-2.55497	2.00199
H	3.70303	-2.78697	1.54072
H	5.32242	-3.41331	1.86448
H	4.51481	-2.42135	3.07599
C	6.57605	-0.91181	2.08345
H	7.27936	-1.74566	2.10998
H	7.06261	-0.06913	1.58845
H	6.35704	-0.62048	3.11395
C	1.61788	3.22132	0.88987
H	2.07469	4.09311	1.36272
H	1.40251	3.45788	-0.15019
H	0.67907	3.02277	1.40479
C	-3.92692	-2.07623	-1.12058
O	-3.95288	-0.79916	-1.18462
O	-4.62465	-2.82313	-1.79892
C	-2.96438	-2.64303	-0.09424
H	-2.54888	-3.58967	-0.44386
H	-3.53325	-2.83297	0.81970
N	-1.90719	-1.67020	0.20719
H	-1.31952	-1.57289	-0.62028
C	-1.05845	-2.13400	1.30066
H	-0.73623	-3.16340	1.10254
H	-1.62399	-2.13882	2.22715
C	0.21221	-1.31922	1.48010
O	0.70738	-1.28380	2.60337
O	0.67033	-0.80207	0.40914
C	-0.37428	0.78543	-1.70710
O	-1.51064	0.21926	-1.56530
O	-0.09486	1.95861	-1.48188
C	0.68902	-0.12181	-2.29440
H	0.61153	-0.06134	-3.38536
H	0.51820	-1.14978	-1.98833
N	2.03118	0.29045	-1.87519
H	2.13268	1.26991	-2.12145
C	3.03905	-0.50892	-2.58957
H	4.00605	-0.00740	-2.53032
H	2.78223	-0.64021	-3.64152
C	3.16344	-1.85656	-1.89282
O	2.89718	-1.82801	-0.64252
O	3.50805	-2.85686	-2.51472
Ru	-2.75892	0.25689	0.13024
C	-2.08881	2.19670	0.76715
C	-1.95293	1.30103	1.83271
C	-3.06963	0.53929	2.27251
C	-4.28186	0.65253	1.56727
C	-4.41041	1.58658	0.50775
C	-3.30723	2.31613	0.03974
H	-1.20382	2.66020	0.35803
H	-0.99032	1.17210	2.30917
H	-5.11474	0.01580	1.83310
H	-5.31302	1.55890	-0.08792
C	-3.38617	3.22372	-1.16204
H	-2.35328	3.43467	-1.44912
C	-4.06417	4.53679	-0.77546
H	-3.54968	5.02014	0.05761
H	-4.06804	5.22656	-1.62143
H	-5.10126	4.36052	-0.47768

C	-4.07052	2.55201	-2.34890
H	-3.99250	3.18367	-3.23530
H	-3.60155	1.59070	-2.56286
H	-5.13261	2.37929	-2.15905
C	-2.96483	-0.37579	3.44889
H	-1.92850	-0.62032	3.67722
H	-3.38754	0.13049	4.31962
H	-3.52876	-1.29581	3.29357

Table S5. Cartesian coordinates and the IR spectrum of [(η^6-*p*-cym)Ru(IdaP)]$^-$ complex.

Electronic Energy (Eh)	-1373.4073006
Sum of electronic and zero-point Energies (Eh)	-1373.087982
Sum of electronic and thermal Energies (Eh)	-1373.066682
Sum of electronic and enthalpy Energies (Eh)	-1373.065738
Sum of electronic and thermal Free Energies (Eh)	-1373.138295
Number of Imaginary Frequencies	0

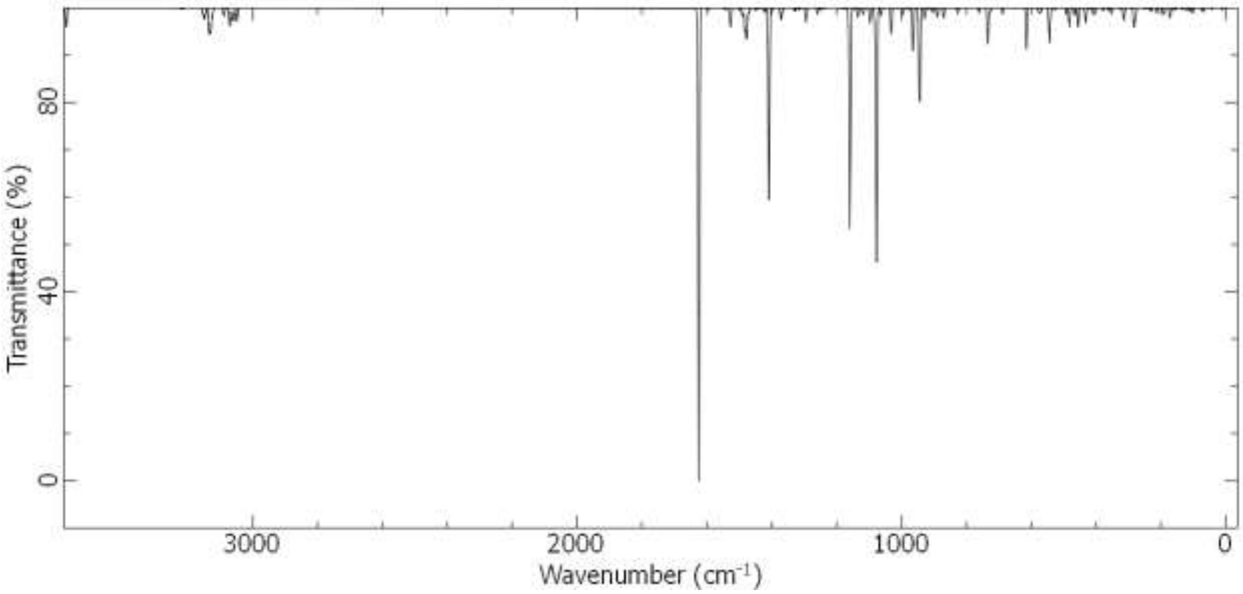

Molecular Geometry in Cartesian Coordinates

Ru	-0.177670	0.464656	-0.043819
C	-1.898849	0.402363	1.298278
C	-1.214890	1.633002	1.440352
C	-0.955633	2.474292	0.335100
C	-1.402170	2.023833	-0.931768
C	-2.094211	0.807102	-1.093985
C	-2.340862	-0.024926	0.026474
H	-2.028104	-0.242669	2.156082
H	-0.799683	1.894510	2.405783
H	-1.124965	2.588370	-1.812848
H	-2.365169	0.484857	-2.087752
C	-3.032814	-1.360664	-0.089705

H	-2.515154	-2.037396	0.594642
C	-2.997260	-1.966010	-1.486070
H	-1.979865	-2.029257	-1.873223
H	-3.411834	-2.974522	-1.452871
H	-3.599698	-1.384733	-2.187665
C	-4.475493	-1.197608	0.397268
H	-4.990868	-2.158851	0.364027
H	-4.512602	-0.824304	1.421981
H	-5.018473	-0.497809	-0.242937
C	-0.182562	3.746215	0.477695
H	-0.865896	4.593066	0.571770
H	0.449475	3.717401	1.364922
H	0.445620	3.913572	-0.397389
C	2.594792	1.083995	-0.841127
O	3.607888	1.370961	-1.487445
O	1.438479	0.936166	-1.359581
C	2.693359	0.965173	0.667504
H	2.729117	1.986541	1.051401
H	3.627841	0.472734	0.936142
N	1.540631	0.283763	1.276356
H	1.311389	0.774046	2.131008
C	1.765785	-1.136401	1.619350
H	0.993800	-1.417097	2.336654
H	2.741304	-1.268062	2.094579
P	1.570306	-2.209395	0.146627
O	1.327715	-3.618185	0.638797
O	2.789433	-2.043002	-0.735423
O	0.291122	-1.593797	-0.493396

Table S6. Cartesian coordinates and IR spectrum of [(η^6-*p*-cym)Ru(Ida2P)]$^{2-}$ complex.

Electronic Energy (Eh)	-1752.1410875
Sum of electronic and zero-point Energies (Eh)	-1751.822104
Sum of electronic and thermal Energies (Eh)	-1751.799509
Sum of electronic and enthalpy Energies (Eh)	-1751.798565
Sum of electronic and thermal Free Energies (Eh)	-1751.873309
Number of Imaginary Frequencies	0

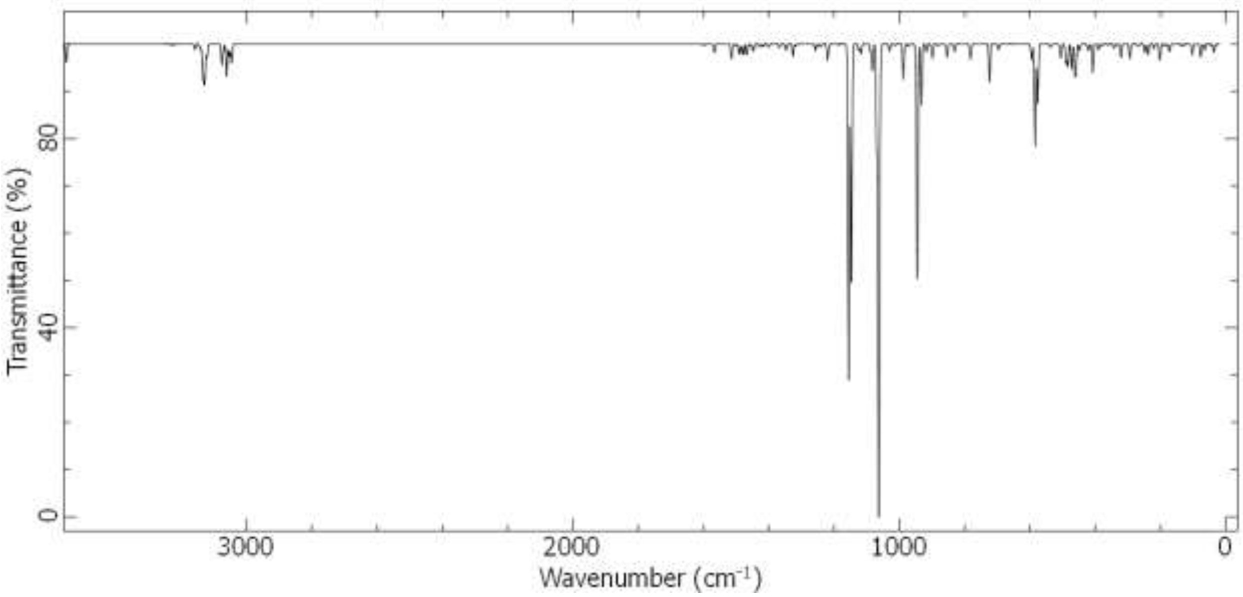

Molecular Geometry in Cartesian Coordinates

Ru	0.196949	-0.354874	0.127983
C	2.082176	-0.039880	1.176613
C	1.184931	-0.660529	2.050829
C	0.525764	-1.873559	1.685871
C	0.813758	-2.422865	0.426735
C	1.741732	-1.799746	-0.456341
C	2.375585	-0.601104	-0.106423
H	2.496533	0.924951	1.437389
H	0.922583	-0.173159	2.980476
H	0.266029	-3.293112	0.091770
H	1.871625	-2.212445	-1.446272
C	3.292594	0.148039	-1.040546
H	3.165149	1.208241	-0.806908
C	2.956660	-0.052981	-2.512272
H	1.903907	0.159327	-2.708805
H	3.562067	0.619758	-3.121513
H	3.169212	-1.073478	-2.838190
C	4.741207	-0.235988	-0.737394
H	5.423334	0.346716	-1.358991
H	4.990187	-0.050361	0.309258
H	4.908221	-1.295581	-0.946748
C	-0.464252	-2.509318	2.606652
H	0.061247	-3.156071	3.313157
H	-1.003145	-1.756449	3.183084
H	-1.180262	-3.114335	2.052164
C	-2.761358	0.458156	0.024763
H	-3.715715	0.486644	0.555931
H	-2.690138	1.355672	-0.589741
N	-1.638988	0.458135	0.986426
H	-1.895811	-0.138372	1.764826
C	-1.323942	1.803844	1.505294
H	-0.711077	1.678468	2.397572
H	-2.239462	2.330688	1.789368

P	-0.358933	2.767727	0.251982
O	0.761756	3.463846	0.997384
O	-1.293377	3.700328	-0.489644
O	0.158611	1.639203	-0.687505
P	-2.606499	-0.983403	-1.088020
O	-2.857138	-2.238473	-0.274186
O	-1.111857	-0.838914	-1.505981
O	-3.533616	-0.787098	-2.264591

Table S7. ESI-MS measured and calculated m/z values of the complexed species registered in the positive mode for the various metal ion - ligand systems at various pH values.

	**m/z measured**	**m/z calculated**	**pH-range**
[(η^5-Cp*)Rh(**Ida**)] + H^+	371.058	371.055	3.06
[(η^5-Cp*)Rh(**Ida**)] + K^+	408.013	408.008	3.06 – 10.11
[(η^5-Cp*)Rh(**Ida**)]$_2$ + H^+	739.104	739.097	2.88 – 3.06
[(η^5-Cp*)Rh(**Ida**)]$_2$ + K^+	777.060	777.053	3.06 – 4.34
[(η^5-Cp*)Rh(**IdaP**H)] + H^+	406.029	406.033	2.36
[(η^5-Cp*)Rh(**IdaP**H)] + K^+	443.988	443.984	2.36 – 10.44
[(η^5-Cp*)Rh(**IdaP**K)]KNO_3 + K^+	582.895	582.892	2.36 – 10.44
[(η^5-Cp*)Rh(**IdaP**H)]$_2$ + H^+	811.057	811.050	2.36
[(η^5-Cp*)Rh(**IdaP**H)]$_2$ + K^+	849.009	849.006	2.36 – 10.44
[(η^5-Cp*)Rh(**Ida2P**H_2)] + H^+	442.009	442.005	2.40 – 4.17
[(η^5-Cp*)Rh(**Ida2P**H_2)] + K^+	479.965	480.005	2.40 – 10.27
[(η^5-Cp*)Rh(**Ida2P**H_2)]KNO_3 + K^+	580.956	580.912	2.40 – 2.57
[(η^5-Cp*)Rh]$_2$(**Ida2P**] + H^+	678.016	678.012	2.40 – 8.21
[(η^5-Cp*)Rh(**Ida2P**H_2)]$_2$ + K^+	920.963	920.959	2.40 – 4.17
[(η^5-Cp*)Ir(**Ida**)] + H^+	460.114	460.110	2.58
[(η^5-Cp*)Ir(**Ida**)] + K^+	498.070	498.065	2.58 – 10.13
[(η^5-Cp*)Ir(**Ida)**]KNO_3 + K^+	599.023	599.017	2.58 – 10.13
[(η^5-Cp*)Ir(**Ida**)]$_2$ + K^+	957.176	957.167	2.58 – 10.15
[(η^5-Cp*)Ir(**IdaP**H)] + H^+	496.091	496.086	2.52
[(η^5-Cp*)Ir(**IdaP**H)] + K^+	534.047	534.042	2.52 – 10.13
[(η^5-Cp*)Ir(**IdaP**)K] + H^+	572.004	571.997	2.52 – 10.13
[(η^5-Cp*)Ir(**IdaP**)K]KNO_3 + K^+	672.956	672.949	2.52 – 10.13
[(η^5-Cp*)Ir(**IdaP**H)]$_2$ + H^+	991.174	991.165	2.52
[(η^5-Cp*)Ir(**IdaP**H)]$_2$ + K^+	1029.130	1029.120	3.76
[(η^5-Cp*)Ir(**Ida2P**H_2)] + H^+	532.069	532.063	2.60 – 4.52
[(η^5-Cp*)Ir(**Ida2P**H_2)] + K^+	570.025	570.018	2.60 – 10.45
[(η^5-Cp*)Ir(**Ida2P**)K_2] + H^+	607.981	607.974	2.60 – 10.45
[(η^5-Cp*)Ir(**Ida2P**H_2)] + H^+	645.936	645.930	4.52 – 10.45

[(η^5-Cp*)Rh(**Ida2P**H_2)]$_2$ + H^+	1063.129	1063.118	2.60 – 2.92
[(η^6-*p*-cym)Os(**Ida**)] + H^+	458.100	458.104	2.63
[(η^6-*p*-cym)Os(**Ida**)] + K^+	496.060	496.056	3.57 –10.44
[(η^6-*p*-cym)Os(**Ida**)]KNO_3 + K^+	597.011	597.007	2.63 –3.57
[(η^6-*p*-cym)Os(**Ida**)]$_2$ + H^+	915.197	915.193	2.63 – 3.57
[(η^6-*p*-cym)Os(**Ida**)]$_2$ + K^+	953.152	953.149	2.63
[(η^6-*p*-cym)Os(**IdaP**H)] + H^+	494.081	494.077	2.45
[(η^6-*p*-cym)Os(**IdaP**H)] + K^+	532.037	532.032	2.45 – 10.38
[(η^6-*p*-cym)Os(**IdaP**K)] + K^+	569.992	569.988	2.45 – 10.38
[(η^6-*p*-cym)Os(**IdaP**H)]$_2$ + H^+	987.150	987.146	2.45
[(η^6-*p*-cym)Os(**IdaP**H)]$_2$ + K^+	1025.106	1025.102	10.38
[(η^6-*p*-cym)Os(**Ida2P**H_2)] + H^+	530.058	530.053	2.44
[(η^6-*p*-cym)Os(**Ida2P**H_2)] + K^+	568.014	568.009	2.44 – 10.45
[(η^6-*p*-cym)Os(**Ida2P**H)K] + K^+	605.970	605.965	2.44 – 10.45
[(η^6-*p*-cym)Os(**Ida2P**)K_2] + K^+	643.925	643.920	4.03
[(η^6-*p*-cym)Os]$_2$(**Ida2P**) + H^+	854.114	854.109	2.44
[(η^6-*p*-cym)Os(**Ida2PH$_2$**)]$_2$ + H^+	1059.104	1059.099	2.44
[(η^6-*p*-cym)Ru(**Ida**)] + H^+	368.041	368.043	2.98
[(η^6-*p*-cym)Ru(**Ida**)] + K^+	405.999	405.999	2.98 – 9.04
[(η^6-*p*-cym)Ru(**Ida**)]$_2$ + K^+	773.032	773.035	6.76 – 9.04
[(η^6-*p*-cym)Ru(**Ida**)]KNO_3 + K^+	506.950	506.951	2.98 – 6.76
[(η^6-*p*-cym)Ru(**IdaP**H)] + H^+	404.012	404.020	2.54 – 3.07
[(η^6-*p*-cym)Ru(**IdaP**H)] + K^+	441.968	441.976	2.54 – 5.60
[(η^6-*p*-cym)Ru(**IdaP**K)] + K^+	479.931	479.932	5.60 – 9.99
[(η^6-*p*-cym)Ru(**IdaP**)]KNO_3 + K^+	580.880	580.883	9.59 – 9.99
[(η^6-*p*-cym)Ru(**Ida2P**H_2)] + H^+	439.986	439.996	2.48
[(η^6-*p*-cym)Ru(**Ida2P**H_2)] + K^+	477.938	477.952	2.48 – 4.09
[(η^6-*p*-cym)Ru(**Ida2P**H)K] + H^+	515.894	515.908	4.09 – 9.80
[(η^6-*p*-cym)Ru(**Ida2P)**K_2] + K^+	553.843	553.864	7.91 – 9.80
